# Attachment Reminders Trigger Widespread Synchrony across Multiple Brains

**DOI:** 10.1523/JNEUROSCI.0026-23.2023

**Published:** 2023-10-25

**Authors:** Ortal Shimon-Raz, Yaara Yeshurun, Adi Ulmer-Yaniv, Ayelet Levinkron, Roy Salomon, Ruth Feldman

**Affiliations:** ^1^Reichman University, Herzliya, 4610101, Israel; ^2^School of Psychological Sciences, Tel Aviv University, Tel Aviv, 69978, Israel; ^3^Sagol School of Neuroscience, Tel Aviv University, Tel Aviv, 69978, Israel; ^4^Department of Cognitive Sciences, University of Haifa, Haifa, 3498838, Israel

**Keywords:** ACC, attachment, intersubject correlation, parental, brain synchrony

## Abstract

Infant stimuli elicit widespread neural and behavioral response in human adults, and such massive allocation of resources attests to the evolutionary significance of the primary attachment. Here, we examined whether attachment reminders also trigger cross-brain concordance and generate greater neural uniformity, as indicated by intersubject correlation. Human mothers were imaged twice in oxytocin/placebo administration design, and stimuli included four ecological videos of a standard unfamiliar mother and infant: two infant/mother alone (*Alone*) and two mother–infant dyadic contexts (*Social*). Theory-driven analysis measured cross-brain synchrony in preregistered nodes of the parental caregiving network (PCN), which integrates subcortical structures underpinning mammalian mothering with cortical areas implicated in simulation, mentalization, and emotion regulation, and data-driven analysis assessed brain-wide concordance using whole-brain parcellation. Results demonstrated widespread cross-brain synchrony in both the PCN and across the neuroaxis, from primary sensory/somatosensory areas, through insular-cingulate regions, to temporal and prefrontal cortices. The *Social* context yielded significantly more cross-brain concordance, with PCNs striatum, parahippocampal gyrus, superior temporal sulcus, ACC, and PFC displaying cross-brain synchrony only to mother–infant social cues. Moment-by-moment fluctuations in mother–infant social synchrony, ranging from episodes of low synchrony to tightly coordinated positive bouts, were tracked online by cross-brain concordance in the preregistered ACC. Findings indicate that social attachment stimuli, representing evolutionary-salient universal cues that require no verbal narrative, trigger substantial interbrain concordance and suggest that the mother–infant bond, an icon standing at the heart of human civilization, may function to glue brains into a unified experience and bind humans into social groups.

**SIGNIFICANCE STATEMENT** Infant stimuli elicit widespread neural response in human adults, attesting to their evolutionary significance, but do they also trigger cross-brain concordance and induce neural uniformity among perceivers? We measured cross-brain synchrony to ecological mother–infant videos. We used theory-driven analysis, measuring cross-brain concordance in the parenting network, and data-driven analysis, assessing brain-wide concordance using whole-brain parcellation. Attachment cues triggered widespread cross-brain concordance in both the parenting network and across the neuroaxis. Moment-by-moment fluctuations in behavioral synchrony were tracked online by cross-brain variability in ACC. Attachment reminders bind humans' brains into a unitary experience and stimuli characterized by social synchrony enhance neural similarity among participants, describing one mechanism by which attachment bonds provide the neural template for the consolidation of social groups.

## Introduction

Cross-brains synchrony research, as indexed by intersubject correlation (ISC) metrics, examines the correspondence in neural activations among individuals when they are exposed to the same dynamic stimulus, typically a movie or a story (Hasson et al., 2004; Nastase et al., 2020; Visconti di Oleggio Castello et al., 2020). Neural synchronization taps the brain's prewired response to the unfolding of events and pinpoints the regions that curate such cross-brains resemblance. Studies on cross-brains mechanisms indicate that similarity in neural response among multiple brains increases when individuals share a narrative (Grall et al., 2021) or view an emotionally arousing movie (Nummenmaa et al., 2012); brains “tick together” when individuals understand a story in the same way (Yeshurun et al., 2017) or become emotionally engaged (Song et al., 2021). It has been theorized that processes of cross-brains concordance played an important role in the evolution of human sociality, providing the foundations for human communication, collaboration, and the capacity for empathy that underpin the formation of social groups and shared cultural experiences (Hasson et al., 2012; Feldman, 2021a). Specifying the processes that sustain the convergent response of multiple brains to ongoing shared events may therefore provide valuable insights into the co-evolution of the social brain and human cultural heritage.

Most cross-brain studies to date presented participants with some form of a narrative (Saalasti et al., 2019; Grall et al., 2021; Nastase, 2021); and, across studies, areas of the default mode network (DMN) have been shown to exhibit cross-brains concordance when individuals process the unfolding of a narrative and integrate it with internal memories or prior knowledge (Yeshurun et al., 2021). Other areas that show cross-brains synchrony are primary sensory regions, higher-order associative areas, such as the superior temporal sulcus (STS) (Hasson et al., 2004), and prefrontal regions (Nguyen et al., 2019); and synchrony in these areas is thought to index the emergence of a shared experience that relies on the similarity of neural response. Cross-brains synchrony in subcortical regions, such as the amygdala or basal ganglia, is less common and has been found in response to emotionally arousing musical stimuli. In these cases, levels of ISC, the main metric for cross-brains synchrony, are linked with moment-by-moment rating of negative valence (Trost et al., 2015), suggesting that fluctuations in cross-brain correlations can track ongoing variability in the emotional features of the stimulus. Multimodal stimuli of musical instrument learning were also found to elicit cross-brains synchrony in paralimbic regions, including the parahippocampal gyrus (PHG) and insula (Fasano et al., 2020), which indicates that activity related to daily living experiences can trigger cross-brain concordance in insular cortex.

Stimuli that represent the mother–infant attachment, whether cues of the parent's own infant or those of a standard unfamiliar infant, have been shown to elicit widespread response in the brains of mothers, fathers, and nonparents across the neuroaxis, from subcortical regions, including the amygdala, VTA, NAcc, and PHG, to paralimbic areas, such as the insula and ACC, and cortical structures, particularly the mPFC (Swain, 2008; Parsons et al., 2013; Abraham et al., 2014; Shimon-Raz et al., 2021). It has been suggested that these areas cohere into the global parental caregiving network (PCN) that supports the formation of the parent–infant attachment (Feldman, 2015, 2017). To trigger response in the PCN, studies used stimuli of an infant alone (Ranote et al., 2004; Noriuchi et al., 2008; Strathearn et al., 2008; Parsons et al., 2017), stimuli of a parent alone during infant-related daily activity (Abraham et al., 2017), or videos of the parent and infant together in various ecological contexts (Kuo et al., 2012; Musser et al., 2012). These “attachment reminders” were found to activate regions of the PCN in studies that presented parents with stimuli of their own infant compared with unfamiliar infant (e.g., Atzil et al., 2011; Abraham et al., 2014) as well as in those that showed a standard unfamiliar infant compared with baseline or control conditions (Mascaro et al., 2014; Parsons et al., 2017). Overall, these findings suggest that any reminder of the parent–infant attachment, whether one's own or generic, induces activation in the parenting network, presumably by activating the parent's “internal working model” of attachment, the representational models that underpin the construction of the parent–child attachment and guide the parent's caregiving role (Bowlby, 1969; Lenzi et al., 2015). Indeed, authors have suggested a partial overlap between areas of the attachment neural system and those of the caregiving network (Lenzi et al., 2015). It is important to note, however, that, while studies have shown activations in nodes of the PCN to both infant–parent alone and parent–infant social stimuli, little attention has been directed to cross-brain synchronization in response to attachment reminders.

As such, the main goal of the current study was to examine whether reminders of the mother–infant attachment, a universal cue that requires no narrative for immediate comprehension, would elicit cross-brains synchronization in areas of the PCN as indexed by ISC, and to test how elaborate is the neural concordance. Second, we wished to estimate the impact of the “social” versus the “alone” attachment context on cross-brain synchronization and gauge whether the degree of neural concordance in response to mother–infant dyadic stimuli (“social”) is greater than to cues of mother or child alone (“alone”). Finally, as oxytocin (OT) is a key modulator of the maternal brain in humans (Numan, 2006; Norholt, 2020) and other mammals (Bosch and Neumann, 2012; Feldman and Bakermans-Kranenburg, 2017; Althammer et al., 2018) and OT administration impacts activity and connectivity in the PCN in response to attachment reminders (Riem et al., 2011b; Bos et al., 2018; Shimon-Raz et al., 2021), we examined the effects of OT administration on cross-brain synchrony in response to four “social” and “alone” attachment stimuli. Two prior studies examined the effect of OT administration on cross-brain synchrony: an EEG study showed enhanced cross-brain synchrony under OT (Mu et al., 2016) and an fMRI study showed that OT administration modulated interbrain correlations in the dorsal DMN and the precuneus network (Wu et al., 2023), and we thus examined the effects of OT on both the PCN and the DMN.

Postpartum mothers, for whom daily mother–infant contexts are the most relevant, rewarding, and arousing (Kim 2016; Parsons et al., 2017), were presented with four movies depicting daily mother–infant “social” and “alone” contexts. Using a double-blind placebo (PBO)-controlled OT-administration crossover design, mothers' brains were imaged twice a week apart. We used a dual analytic approach to examine cross-brains synchronization to attachment reminders; “theory driven” and “data-driven.” The “theory driven” approach was based on multiple imaging studies that pinpointed nodes of the PCN; and these were preregistered, along with the DMN, as our ROIs and were expected to show above-threshold cross-brain synchronization to attachment cues. Special attention was paid to the preregistered insula and ACC regions, which showed greater responsivity to own mother–infant stimuli compared with unfamiliar mother–infant stimuli in a previous study with the same participants (Shimon-Raz et al., 2021). As the insula and ACC curate features of the mother-own-infant attachment, we explored whether they also take part in processing cross-brains synchronization to general attachment reminders and play a role in sustaining moment-by-moment variability in cross-brains synchronization. The “data-driven” analysis used resting-state-based parcellation to examine ISCs across the entire brain (Shen et al., 2013). These methods combined a top-down with a bottom-up approach to define areas that exhibit cross-brain synchronization to attachment reminders.

Three hypotheses were preregistered (https://osf.io/bmp43?view_only=0ca4cb28ef2c4b4092a44a1c047c9242). First, we hypothesized that a synchronized response, as measured by ISCs, in brain regions of the PCN and DMN would be found when mothers observe ecological videos of attachment contexts (i.e., attachment reminders). Because a mother's brain is selectively wired to social moments (Shimon-Raz et al., 2021), our second hypothesis contended that the social contexts would elicit greater cross-brains synchronization in the PCN compared with contexts of mother or infant alone. Finally, as OT is a key modulator of the maternal brain (Panksepp et al., 1997; Marlin and Froemke, 2017; Sanson and Bosch, 2022), we hypothesized that OT would modulate cross-brains synchronization levels in postpartum mothers. In addition to these preregistered hypotheses, we also examined whether modulations in behavioral synchrony during the free play vignette will be tracked by modulations in mothers' cross-brains synchronization. The ACC and insula play a key role in the neural representation of attachment (Ulmer-Yaniv et al., 2022), and show greater activation to “own” mother–infant attachment stimuli (Shimon-Raz et al., 2021). We therefore expected that cross-brains synchronization fluctuations in the ACC and insula may track fluctuations in the level of mother–infant behavioral synchrony in the presented video. Overall, a widespread cross-brains synchronization, if found, would suggest that any reminder of the mother–infant attachment, whether one's own or generic, induces substantial concordance across multiple brains and reduces neural variability. Such findings may expand our understanding on the neural origins of social-group formation in humans.

## Materials and Methods

### Participants

The initial sample included 35 postpartum mothers who were recruited through advertisements in online parenting forums. Following recruitment, mothers underwent a brief phone screening for MRI scanning and postpartum depression using the Edinburgh Postnatal Depression Scale (EPDS) (Cox et al., 1987). Cutoff for joining the study was EPDS score of ≤8 (score >9 indicates minor depression). Next, mothers were invited to a psychiatric clinic for a psychiatric evaluation before the scanning sessions. During this visit, mothers were interviewed using the Structured Clinical Interview for the DSM-IV to assess current and past psychiatric disorders. None of the participants met criteria for a major or minor depressive episode during the perinatal period, 97% did not meet criteria for any diagnosable psychopathology during this time, and 86% did not meet criteria for any diagnosable psychopathology disorder during their lifetime. All participants were married, cohabitated with the infant's father, were considered to be of middle-or upper-middle socio-economic status, and completed high school and at least some college.

Of the 35 participants, three did not complete a single scan (one because of medical problems and two because of claustrophobia). After examining the quality of the data, four mothers were excluded because of excessive head movement artifacts (movements ≥3 mm). In an additional participant, we identified unexplained noise in the signal, detected by contrasting the visual conditions versus rest. Three other mothers watched a different set of stimuli and were unable to enter the ISC analysis. These 11 subjects were removed before analysis of the experimental effects.

The final sample used for the analysis included 24 mothers (mean age = 29.62 years, SD = 4.9 years; EPDS mean score = 3.12, SD = 2.50) of 4- to 7-month-old infants (mean age = 5.58 months, SD = 1.37 months) and each mother underwent scanning twice (48 scans). A sample size of 24 participants has been shown in previous studies to be sufficient for assessing neural responses to naturalistic stimuli (Ames et al., 2015; Yeshurun et al., 2017) and for power analyses of ISC, the analytic method in the current study (Pajula and Tohka, 2016).

The study was approved by the Helsinki committee of the Sourasky Medical Center, Tel Aviv (Ethical approval 0161-14-TLV). All participants signed an informed consent. Subjects received a gift certificate of 700 NIS (∼$200 U.S.) for their participation in all four phases of the study (diagnosis, home visit, and two imaging sessions).

### Stimuli and experimental design

Following psychiatric evaluation, the study included three sessions for each mother. In the first, families were visited at home and episodes of mother–infant free-play interaction, infant alone, mother alone, and breastfeeding were videotaped. In addition, mothers completed self-report measures.

In the second and the third sessions, mothers participated in fMRI brain scanning at the Tel Aviv Sourasky Medical Center. Before each scan, mothers received 24 IU of PBO or OT intranasally in a randomized, PBO-controlled, double-blind, two-period crossover design. On average, 14 d elapsed between the two scans (SD = 9.30, mode = 7, median = 7), that were both scheduled for the morning hours (07:30-12:00).

**Figure 1.**
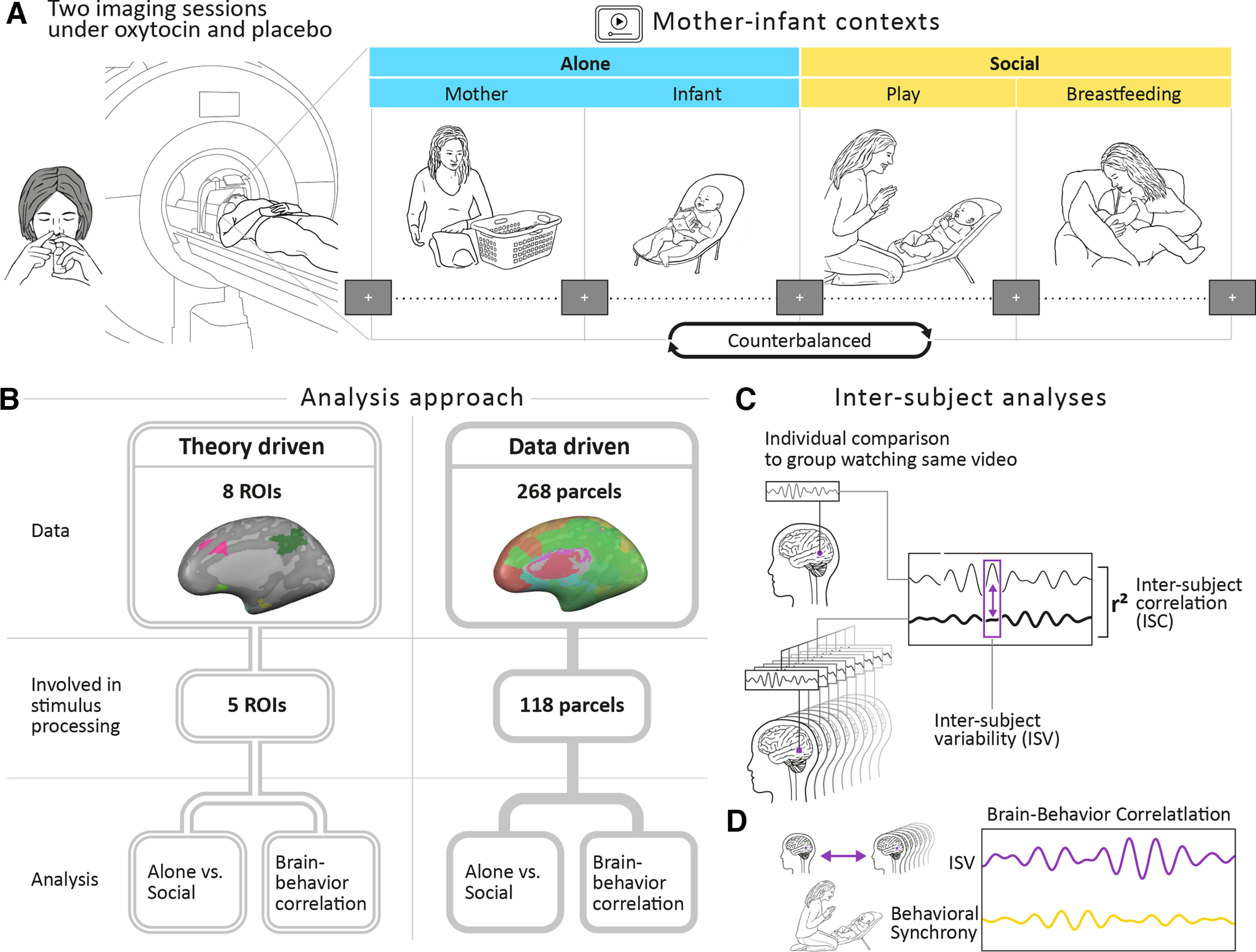
Research plan. ***A***, Experimental procedure and paradigm. Postpartum mothers were imaged twice after OT/PBO administration in a randomized, PBO-controlled, double-blind, crossover design. On average, 2 weeks elapsed between scans. While in the scanner, participants observed four daily ecological video vignettes showing a standard mother and an infant apart and together. The *Alone* context condition (blue) included two videos of the mother alone while folding infant laundry and of the infant alone sitting in the bassinet while playing with a toy. In the *Social* context condition (yellow), two videos showed the mother and the infant together while engaged in free-play interaction and during a breastfeeding episode. Videos lasted 2 min each and were previewed by rest with fixation period of 1 min. A rest with fixation periods of alternately 15-18 s was presented between clips. Order of videos was counterbalanced between the two scans. Bayesian ANOVA results of two conditions are presented in Extended Data Figures 1-1 and 1-2. ***B***, Two approaches were used to examine differences in ISC: a theory-driven approach focused on 8 preregistered ROIs, 7 within the PCN and the DMN (Extended Data Fig. 1-3); and a data-driven approach that tested the entire brain using Shen et al. (2013) atlas that consist of 268 parcels. In the two approaches, we first calculated ISC value for each brain area and compared it with ISC threshold. Regions showing ISC above the null distribution threshold were defined as involved in stimulus processing. In these areas, we explored differences between the ISC in the Alone and the Social conditions, and the correlation between the participants' moment-by-moment neural similarity and the continuous level of mother–infant behavioral synchrony in the free-play interaction. ***C***, ISC measures the neural similarity across participants by correlating the time course of one participant with the averaged time course of all other participants in the same brain region. ISV is a complimentary measure that reflects the neural variability of the group in a certain time point. It was calculated using ED, which is the difference in the BOLD signal at a certain time between a subject and the rest of the group's mean. ***D***, Brain–behavior correlation was examined in response to the free-play interaction video. In each TR mother–infant, behavioral synchrony was evaluated using a micro-coding system and correlated with the continuous time course of the ISV. ISV score was transformed to *z* score and multiplied by −1 in order that higher values will represent greater neural/behavioral synchrony. For detailed description of the behavioral coding, see Extended Data Figure 1-4.

The mother–infant context paradigm and fMRI sequence began ∼30 min after intranasal PBO/OT administration. During the scans, each mother was presented with 8 naturalistic films of 120 s each: four videos of the same unfamiliar mother and her infant, and four individually tailored matched videos of the mother herself and her own infant. In this paper, only the neural response to the standard-unfamiliar mother and infant videos was analyzed and is therefore referred as our two stimuli. The *Alone* stimulus included two videos: one of the infant alone (in the bouncer, playing with a toy) and one of the mother alone (sitting, folding laundry); while the *Social* stimulus included two videos of the mother and the infant together: one during a free-play (playing together while the infant is in the bouncer) and one during breastfeeding. Between videos, a fixation of a black cross over a gray background was presented. Fixation duration alternated between 15 and 18 s. The order of the videos was counterbalanced across participants and scans; while in the scanner, mothers were asked to watch the movies attentively. Video clips were played using VLC media-player (version 2.2 for Windows, VideoLAN). Study procedure and fMRI paradigm are presented in Figure 1*A*.

10.1523/JNEUROSCI.0026-23.2023.f1-1 Figure 1-1 *2* × *2* Bayesian repeated-measures ANOVA results (*Infant*/*Mother alone* video× *PBO-OT*) demonstrated evidence for the lack of difference between the 2 *Alone* context videos ISCs, between *PBO* and *OT* and the lack of interaction effect between them, through the PCN. Abbreviations: OT, Oxytocin; PBO, Placebo; PCN, Parental Caregiving Network. Download Figure 1-1, DOCX file.

**Figure 2.**
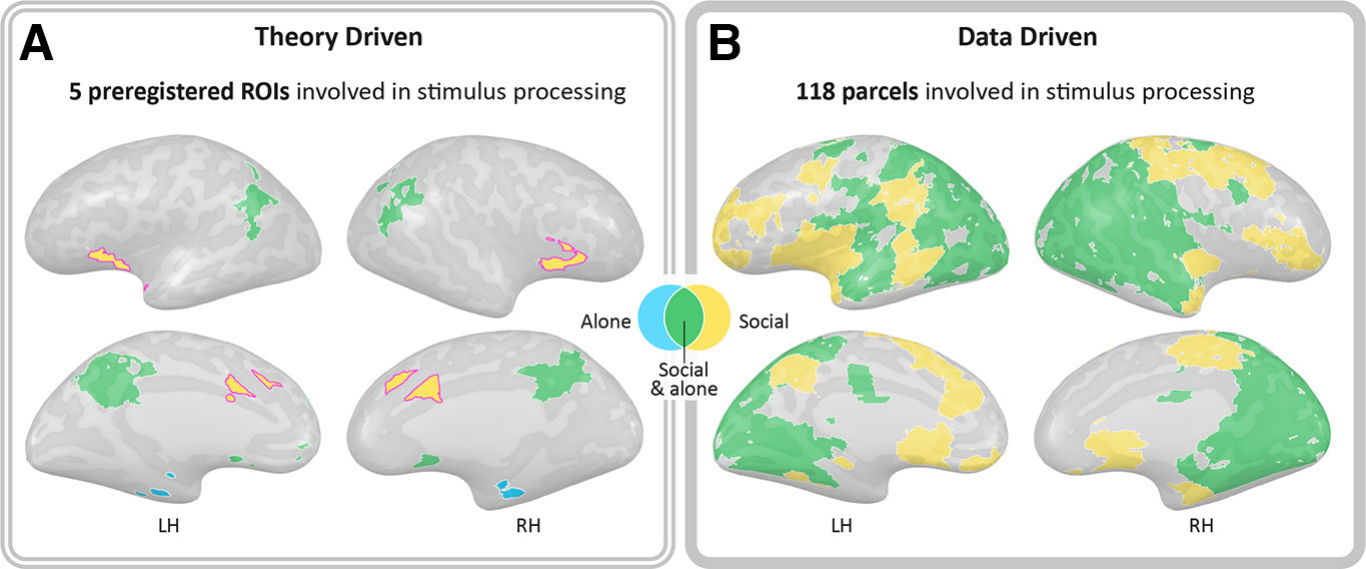
Brain regions involved in stimuli processing (significant ISC > ISC threshold, FDR-corrected). Yellow represents areas that responded to the *Social* context. Blue represents areas that responded to the *Alone* context. Green represents areas that responded to both *Social* and *Alone* stimuli. ***A***, Examination of the 8 preregistered ROIs revealed five areas that yielded high ISC, including four areas of the PCN and the DMN. The preregistered insula and ACC responded to the *Social* context with high ISC. These are self-attachment regions of the PCN that respond selectively to self-related stimuli. The PHG responded to the *Alone* context videos; the NAcc and the DMN responded to both conditions. ***B***, A total of 118 of 268 parcels showed high ISC in response to the stimuli (Extended Data Fig. 2-1). Along these, areas ranging from the subcortical striatum, insula, ACC, NAcc, PHG, to the STS, TPJ, premotor, visual, auditory, and somatosensory cortices, the OFC and prefrontal areas; 75 parcels responded to both stimuli; 42 responded exclusively to the *Social* condition; and 1 parcel, in the cerebellum, responded only to the *Alone* condition. This highlights extensive similarity in neural response to attachment-related stimuli, particularly to the *Social* context. Similar areas were elicited in both analysis approaches. ISC thresholds are presented in Extended Data Figures 2-2 and 2-3. TPJ, Temporoparietal junction. Pink outline indicates self-attachment ROIs.

10.1523/JNEUROSCI.0026-23.2023.f1-2 Figure 1-2 *2* × *2* Bayesian repeated-measures ANOVA results (*Free-play*/*Breastfeeding* video × *PBO/OT*) revealed the lack of difference between the 2 *Social* context videos ISCs between *PBO* and *OT* and the lack of interaction effect between them, through the PCN. Abbreviations: OT, Oxytocin; PBO, Placebo; PCN, Parental Caregiving Network. Download Figure 1-2, DOCX file.

**Figure 3.**
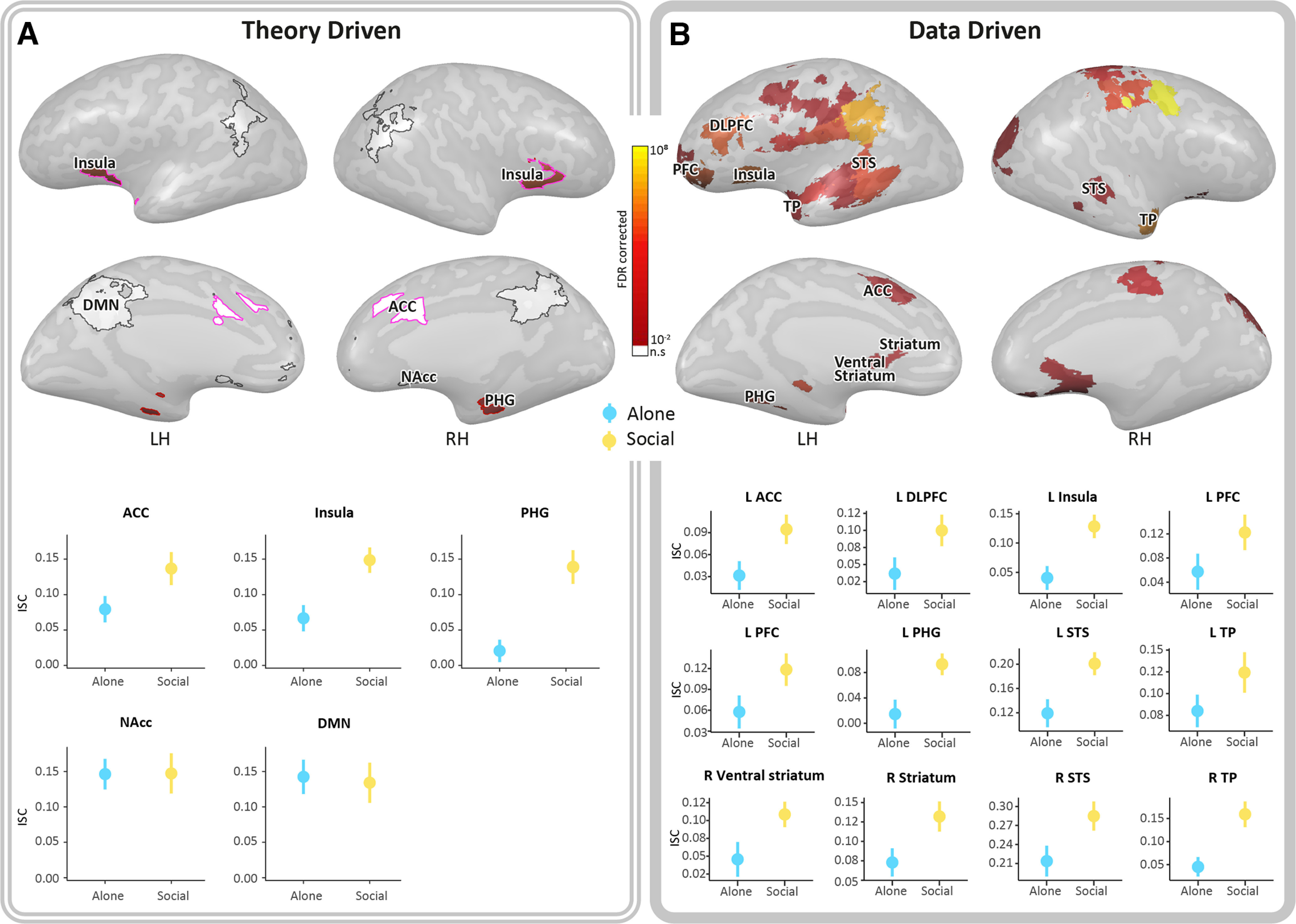
Significant main effect of *Context* was driven by greater ISC under the *Social* condition compared with the *Alone* condition. All results are FDR-corrected. ***A***, A repeated-measures ANOVA (*ROI* × *Context* × *PBO-OT*) revealed significant main effect of *Context* that was driven by higher ISC under the *Social* context compared with the *Solitary* context in the preregistered insula and PHG (red) (Extended Data Figs. 3-1 and 3-2). ROIs in white represent areas that responded to the stimuli but did not show such main effect. Pink outline indicates ROIs that are selectively responsive to self-attachment stimuli. Graphs represent ISC means in the *Social* (yellow) and in the *Alone* (blue) conditions. Error bars indicate SEM. ***B***, Whole-brain results of 2 × 2 repeated-measures ANOVA (*Context* × *PBO-OT*), conducted for each of the 118 parcels involved in stimulus processing, revealed 27 parcels in which neural similarity was higher under the *Social* condition (Extended Data Fig. 3-3). Within them, 12 areas of the PCN that overlap with our ROIs and with Neurosynth map of the term “social” (Extended Data Fig. 3-4). In the left hemisphere: ACC, insula, DLPFC, PFC, PHG, STS, and TP. In the right hemisphere: striatum and VS, STS, and TP. The level of significance of each region is indicated by a color scale ranging from red to yellow. Additional areas found include the OFC, motor, auditory, and somatosensory cortices whose graphs are shown in Extended Data Figure 3-5. TP, Temporal pole; VS, ventral striatum; RH, right hemisphere; LH, left hemisphere.

10.1523/JNEUROSCI.0026-23.2023.f1-3 Figure 1-3 Eight preregistered ROIs, including the ACC, amygdala, insula, NAcc, PHG, TP, VTA in the PCN (upper), and the DMN (footer). Abbreviations: ACC, anterior cingulate cortex; DMN, default mode network; NAcc, nucleus accumbens, PCN, parental caregiving network; PHG, parahippocampal gyrus; TP, temporal pole; VTA, ventral tegmental area. Download Figure 1-3, TIF file.

**Figure 4.**
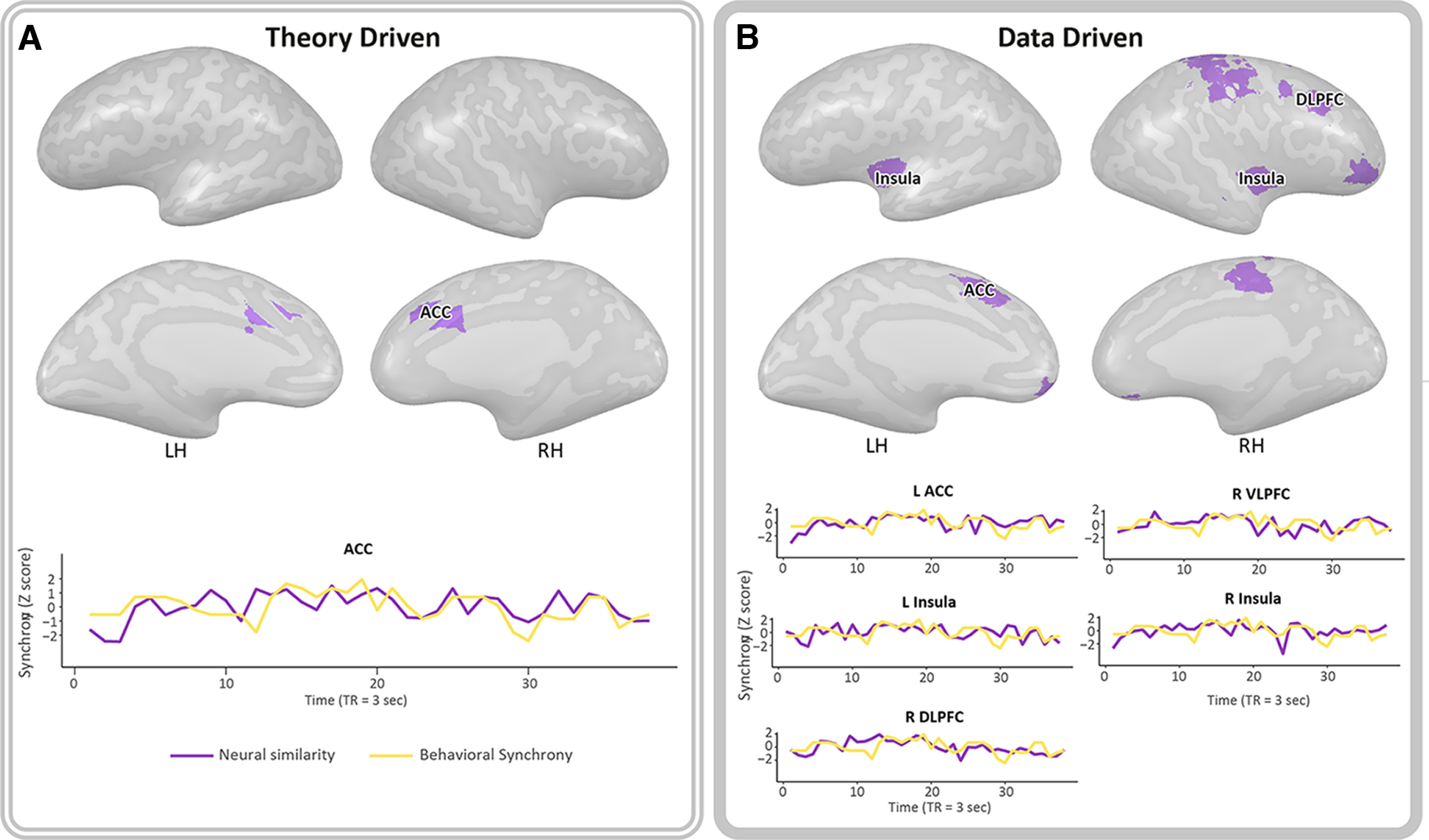
Brain–behavior correlations. The figure represents brain areas where the level of cross-brain synchrony was correlated with mother–infant behavioral coordination in the free-play video that was viewed. ***A***, Significant Pearson's correlation showed that, in the preregistered ACC, moments of greater behavioral synchrony were related to greater neural similarity between subjects (nonsignificant results are in Extended Data Fig. 4-1). This result remains significant after multiple comparisons correction. The preregistered ACC responds selectively to self-attachment stimuli in similar paradigm within the same sample. Purple line in the graph indicates neural similarity calculated by *z* transformation of the 1 – ISV score. Yellow line indicates the level of mother–infant behavioral synchronization based on micro-coding of the mother's and infant's gaze, affect, and touch in each TR. ***B***, Data-driven analyses of Pearson's correlations identified 11 parcels who showed significant positive brain–behavior correlation that did not survive correction for 42 comparisons. Among these, areas of the PCN: the left ACC, which overlap with the preregistered ACC, right and left insula, right VLPFC and right DLPFC. Their graphs are shown in the figure. Other areas that were found include the motor, primary motor, and somatosensory cortices, left and right OFC, and the cerebellum (Extended Data Fig. 4-2). VLPFC, Ventrolateral PFC; RH, right hemisphere; LH, left hemisphere.

10.1523/JNEUROSCI.0026-23.2023.f1-4 Figure 1-4 Behavioral coding of mother–infant synchronization during *Free-play* interaction. Behavior was coded over 3 non-verbal parameters: *gaze*, *affect*, and *vocalization*. The synchronization level indicates the degree of compatibility between the mother and the baby, with a higher score indicating a higher level of behavioral synchronization. Download Figure 1-4, DOCX file.

### MRI acquisition

MRI data were collected using a 3T scanner (Siemens Magnetom Prisma syngo MR D13D) located at the Tel Aviv Sourasky Medical Center. Scanning was conducted with a 20-channel head coil for parallel imaging. Head motion was minimized by padding the head with cushions, and participants were asked to lie still during the scan. High-resolution anatomic T1 images were acquired using MPRAGE sequence: TR = 1860 ms, TE = 2.74 ms, FOV = 256 mm, voxel size = 1 × 1 × 1 mm, flip angle = 8°. Afterward, functional images were acquired using EPI gradient echo sequence. TR = 3000 ms, TE = 35 ms, 44 slices, slice thickness = 3 mm, FOV = 220 mm, voxel size = 2.3 × 2.3 × 3 mm^3^, flip angle = 90°. In total, 381 volumes were acquired over the course of the context paradigm. Visual stimuli were displayed to subjects inside the scanner, using a projector (Epson PowerLite 74C, resolution = 1024 × 768), and were back-projected onto a screen mounted above subjects' heads, and seen by the subjects via an angled mirror. The stimuli were delivered using Presentation software (www.neurobs.com).

### OT administration

Mothers were asked to self-administer 24 IU of either OT (Syntocinon Nasal spray, Novartis; three puffs per nostril, each containing 4 IU) or PBO before scanning. The PBO was custom designed by a commercial compounding pharmacy to match drug solution without the active ingredient. The same type of standard pump-actuated nasal spray was used for both treatments.

### Behavioral data analysis

#### Micro-coding of social synchrony during mother–infant free-play condition

To track the variability in the degree of behavioral synchrony during the free-play film, we applied the *Parent–Infant Synchrony* (Feldman and Eidelman, 2007) coding scheme to the free play session, consistent with our previous research (Feldman and Eidelman, 2003, 2007; Feldman et al., 2004), including fMRI studies (Atzil et al., 2011; Shimon-Raz et al., 2021). Micro-coding was performed by a trained coder on a computerized system (Mangold-Interact, RRID:SCR_019254) in 3 s frames. Three nonverbal aspects of social behavior were coded for mother and infant separately: *Affect* (very positive, positive, neutral, negative withdrawn, negative/upset, uncodable), *Gaze* (infant: to mother, to object, joint gaze to object, to environment, sleepy/drowsy, aversion, uncodable; mother: to infant's face, to infant's body, to object, joint gaze to object, to environment, aversion, uncodable), and *Vocalization* (infant: cooing, fussing, laughing, crying, no vocalization, uncodable; mother: motherese, adult directed speech to infant, adult speech to another adult, laugh, no vocalization, uncodable). Synchrony was defined, consistent with our prior research (Feldman and Eidelman, 2007), by conditional probabilities (infant in State A given mother in State A), indicating episodes when the mother and the infant were both in social gaze, positive vocalizations, and shared positive affect (Feldman and Eidelman, 2007; Granat et al., 2017). The degree of synchrony was graded on a scale from 1 to 15. The lowest level of synchrony (coded as level 1) involved mother looking at infant with neutral affect and no vocalization and infant gaze-averting and expressing no positive affect or vocalization. The highest level of synchrony (coded as level 15) involved mother looking at infant, expressing positive affect, and vocalizing while infant looked at mother's face, was positive, and laughed.

### MRI data analysis

#### Data preprocessing

Data preprocessing and data analysis were conducted using BrainVoyager QX software package 20.6 (Brain Innovation, RRID:SCR_013057) (Goebel et al., 2006). The first three functional volumes, before signal stabilization, were automatically discarded by the scanner to allow for T1 equilibrium. Preprocessing of functional scans included 3D motion correction, slice scan time correction, spatial smoothing by a FWHM 6 mm Gaussian kernel, and temporal high-pass filtering. The functional images were then manually aligned and coregistered with 2D anatomic images and incorporated into the 3D datasets through trilinear interpolation. The complete dataset was normalized into MNI space (Evans et al., 1994).

The scanning sessions resulted in 40-TR-long recordings of BOLD signal intensity per film, from which the first 2 TRs (equivalent to 6 s) were excluded, to consider hemodynamic response. ROI and parcel-specific BOLD time course were produced for each subject and video, by averaging the time-series of all voxels in the same area. *Z* scores were calculated for each time series separately, giving each TR a normalized value representing signal intensity

#### ROI preregistration and analysis

ROI analysis was conducted on eight preregistered bilaterally defined ROIs, seven of which marked as the “maternal caregiving network” and the DMN. PCN regions included the ACC and the insula, which were previously shown to be preferentially activated to self-stimuli (Shimon-Raz et al., 2021) as well as the amygdala, hippocampus/PHG, temporal pole, VTA, and NAcc. ROIs were selected *a priori*, based on theory and literary meta-reviews (Abraham et al., 2016; Lindquist et al., 2016), and on a pilot study of 4 subjects that completed similar paradigm and were not included in the current study. ROIs were defined functionally and anatomically, verified and validated by human brain database platforms: Talairach Daemon (Lancaster et al., 2000) and Neurosynth (Yarkoni et al., 2011), registered at the Open Science Framework before data analysis and transformed into MNI space.

Time courses were extracted from ROIs, and ISC for each ROI was calculated. Five ROIs, four of the PCN and the DMN, passed the ISC null distribution threshold. Within these regions, we analyzed the data with two separate repeated-measures ANOVA. In the PCN, a 4 × 2 × 2 (*ROIPCN* × *Context* × *PBO-OT*) was computed; and in the DMN, a 2 × 2 (*Context* × *PBO-OT)* was calculated, thus allowing to investigate main effects of stimulus type, OT administration, and their interactions. To further examine the origin of main effects and interactions, simple effect analyses and False Discovery Rate (FDR)-corrected *post hoc* tests were conducted.

#### Data-driven whole-brain analysis

In the current study, we used ISC to identify regions that were involved in processing the video-clips. We conducted both whole-brain analysis using Shen's parcellation (Shen et al., 2013) and ROI analysis on predefined areas. Shen's whole-brain parcellation defines 268 parcels based on resting-state fMRI data that yielded nodes with coherent internal time courses (Shen et al., 2013). In the current study, parcels were labeled with their serial number, which ranged from 1 to 268 in the atlas, as well as their location association in meta-analysis maps in the Neurosynth human brain database platform.

ISC and *p* values were calculated for the four conditions (*Alone/Social* × *PBO/OT*) in each parcel. Next, ISC null distribution thresholds and FDR-corrected *p* values were calculated. In parcels that passed the threshold, a 2 factor (*Context* × *PBO-OT*) repeated-measures ANOVA was performed and yielded 2 main effects and an interaction effect for each parcel.

#### ISC

To test our preregistered hypothesis that *Social* videos would yield greater neural similarity among subjects than *Alone* videos, we compared both conditions' ISC, a measure of neural response coherence across individuals over the time course of a stimulus.

We calculated an ISC score for a given brain region (ROI/parcel) by correlating each participant's time course with the averaged time course of the other participants using Pearson's correlation. This procedure resulted in 24 ISC values (one per participant), which were averaged to obtain one ISC value per region. Higher ISC imply more synchronized brain responses to the stimuli.

Analysis of effects within a 2 factors Bayesian repeated-measures ANOVA (*Infant/Mother* alone movie × *PBO-OT*) showed moderate evidence for the absence of difference between the mother alone and the infant alone movies (*BF*_10_ = 0.25, *BF*_10incl_ = 0.18), and similar analysis that was computed and revealed moderate evidence against the difference between the play and the feeding movies (*BF*_10_ = 0.341, *BF*_incl_ = 0.246). For testing the response to *Alone* versus *Social* stimuli, time courses sampled during the presentation of the infant alone video and the mother alone video, 38 TRs each, were concatenated into a 76-TR-long sequence of an *Alone* condition, while time courses of the *breastfeeding* and the mother–infant *free-play* videos were concatenated into a *Social* condition sequence. Next, the two 76 TR sequences had their ISC coefficient calculated separately.

The structure of a subject w's joined time-series at a certain ROI or parcel as follows:

$$S_{Social}\left(w\right)=\left\{{\text{S}}_{Play,1}\left(w\right),...,S_{Play,38}\left(w\right),S_{Feeding,1}\left(w\right),...,S_{Feeding,38}\left(w\right)\right\}$$

$$S_{Alone}(w)=\left\{S_{Infant,1}\left(w\right),...,S_{Infant,38}\left(w\right),S_{Mother,1}\left(w\right),...,S_{Mother,38}\left(w\right)\right\}$$

ISC score significance was tested using bootstrapping. A resampling procedure was applied to each of the *Alone* and *Social* sequences for PBO/OT separately. FFT was used to separate each sequence into independent components while preserving the power spectrum of the signal. Then, 10,000 randomized phase permutations of the sequences were assembled using inverse FFT. By calculating each of the new time course's ISC, we achieved a null distribution of 10,000 ISC scores.

In each condition, given an area (either ROI or parcel) with an average ISC score of $\bar{M}$, the *p* value for that area was determined by the proportion of ISC scores greater than $\bar{M}$ in the null distribution. Adding one to the count of said values ensures *p* is a non-zero probability as follows:

$$p\left(\bar{M}\right)=\frac{\left|\left\{r|r\in ISC_{Null},r>\bar{M}\right\}\right|+1}{10,000}$$

Critical *p* values were calculated using Benjamini–Hochberg's correction for controlling the FDR (Benjamini and Hochberg, 1995) as follows:

- Sort the obtained *p* values: $\left\{{\text{p}}_{1},...,{\text{p}}_{\text{m}}\right\}$ (${\text{p}}_{i}$ denotes the i-smallest *p* value).
- For i={1,...,m}, calculate ${\text{p}}_{crit}\left(i\right)=\frac{\text{i}\alpha }{\text{m}}$ (where *m* is the number of comparisons and α is the probability threshold; in our case, *m* = 2 × 8 = 16, α = 0.05).
- Let *k* be the largest i∈[1,m] such that ${\text{p}}_{i}\leq {\text{p}}_{crit}(i)$. Then the critical *p* value is ${\text{p}}_{crit}\left(\text{k}\right)$.

To narrow down all tested regions to those that yielded the strongest coherence, an additional (more selective) threshold was applied. This threshold was the ISC score of the upper fifth percentile of the null distribution of each condition. Brain areas that passed that null distribution threshold were defined as involved in stimulus processing and continued to ANOVA, as described later in Results.

#### Intersubject variability (ISV)

ISV is a measure of the magnitude of dispersion in ISC scores within the group at a certain time. The dispersion was determined using Euclidean distance (ED), which is the difference in the BOLD signal magnitude at a certain time between a subject and the rest of the group's mean. The ED of subject *s* at TR *t* can be calculated by the following expression:

$$\text{ED}\left(s,t\right)=S_{s,t}-\frac{\sum_{i=0,i\neq s}^{n}S_{i,t}}{n-1}$$

Where *n* is the number of subjects (24) and $S_{i,t}$ is the value of the *t*^th^ sample in the normalized time course of subject *i*.

ISV was calculated for the *Social* free-play condition in each ROI and parcel that were involved in stimulus processing (passed the ISC null distribution threshold) by averaging the squared ED values of each TR (between 24 subjects), then dividing by the greatest ED among all TRs in the time series corresponding to the *free-play* film.

(1) $$\text{ ISV}\left(t\right)=\frac{\bar{ED^{2}\left(t\right)}}{\max ED^{2}}$$

(2) $${\left\{{\text{ISV}}_{\text{Play}}\left(t\right)\right\}}_{t=1}^{38}$$

In order to quantify the similarity in responses instead of dispersion, we applied the following transformation for the resulting ISV time-series (Eq. 2):

(3) $${\left\{1-{\text{ISV}}_{\text{Play}}\left(t\right)\right\}}_{t=1}^{38}$$

Next, Pearson's *r* was calculated between the series of 38 average *Z* scores (one for each TR) in the ISV complement time series (described in Eq. 3), and the series of behavioral synchrony *Z* scores at each TR in the *free-play* video.

### Statistical analysis

For statistical analysis, we used JASP (version 0.9.2.0 for Windows, JASP Team, 2018, RRID:SCR_015823), SPSS (SPSS statistics version 25.0, IBM) and R software (version 3.5.3, R Core Team, 2017, RRID:SCR_019096). For all ISC calculations, we used MATLAB (version 2021b, The MathWorks).

## Results

### Brain regions showing cross-brain synchrony to attachment reminders

Consistent with our first preregistered hypothesis, daily ecological mother–infant contexts induced above threshold intersubject brain synchronization in the PCN and in the DMN. Four of the 7 preregistered ROIs of the PCN as well as the DMN showed high ISC for at least one context under PBO or OT (all *p* values < 0.001). The ACC and the insula responded exclusively to the *Social* context videos (and not to the *Alone* videos), the PHG responded to the *Alone* context, and the NAcc and the DMN were involved in both contexts processing (Fig. 2*A*).

10.1523/JNEUROSCI.0026-23.2023.f2-1 Figure 2-1 ISC scores of the parcels involved in stimulus processing in one condition or more. Only above-threshold values are displayed. Download Figure 2-1, DOCX file.

10.1523/JNEUROSCI.0026-23.2023.f2-2 Figure 2-2 ISC scores and *p* values of the predefined ROIs, and threshold values for each condition in the theory-driven analyses. Results are FDR-corrected. Abbreviations: OT, oxytocin; PBO, Placebo; *, ROIs with above-threshold ISC under at least one condition. Download Figure 2-2, DOCX file.

10.1523/JNEUROSCI.0026-23.2023.f2-3 Figure 2-3 ISC threshold values for each condition in the data-driven analyses (parcels). Download Figure 2-3, DOCX file.

Furthermore, whole-brain ISC revealed 118 parcels that were involved in processing the *Alone* and/or *Social* contexts videos, including areas in the PCN and the DMN. Of these parcels, 75 parcels were involved in processing of both *Social* and *Alone* conditions. These regions included areas in the PFC, posterior insula, hippocampus, PHG, cuneus and precuneus, fusiform gyrus, temporoparietal junction, inferior parietal, premotor, visual, auditory and somatosensory cortices, and areas in the cerebellum (Fig. 2*B*). Forty-two parcels were only involved in processing the *Social* context, including areas in the PFC, orbitofrontal, superior temporal and premotor cortices, as well as subcortical striatum and the NACC, insula, ACC and hippocampus; and one parcel in the cerebellum was only involved in processing the *Alone* condition.

### *Social* versus *Alone* contexts

The second preregistered hypothesis suggested increased ISC in the PCN in response to videos depicting mother–infant contexts compared with mother alone and infant alone videos.

For the four ROIs involved in stimulus processing within the PCN (ACC, insula, PHG, and NAcc), a repeated-measures ANOVA (*ROI* × *Context* × *PBO-OT*) revealed significant main effect of *Context*. The *Social* context elicited higher ISC compared with the *Alone* context [*F*_(1,23)_ = 10.96, *p* < 0.01, Eta^2^ = 0.32], (Mean*_Social_* = 0.14, SE*_Social_* = 0.07; Mean*_Alone_* = 0.08, SE*_Alone_* = 0.06). Additionally, a significant main effect for *ROI* was found with the NAcc yielding higher ISC compared with the PHG [*t*_(23)_ = 3.28, p_bonf_ = 0.02; Mean_NAcc_ = 0.15, SD_NAcc_ = 0.09_;_ Mean_PHG_ = 0.07, SD_PHG_ = 0.06). Interaction effects were not significant. *Post hoc* repeated-measures ANOVA (*Context* × *PBO-OT*) conducted in each of the ROIs revealed significant *Context* main effect in the insula and in the PHG, both driven by greater ISC in response to the *Social* context compared with the *Alone* context (Mean*_Social-Insula_* = 0.15, *SD Social_-Insula_* = 0.08, Mean*_Alone-Insula_* = 0.07, SD*_Alone-Insula_* = 0.09; Mean*_Social-PHG_* = 0.12, SD*_Social-PHG_* = 0.11, Mean*_Alone-PHG_* = 0.02, SD*_Alone-PHG_* = 0.08) (Fig. 3*A*). No such effect was found in the ACC and the NAcc. In the DMN, which was also involved in stimulus processing, 2 factorial repeated-measures ANOVA (*Context* × *PBO-OT*) did not reveal any significant effects.

10.1523/JNEUROSCI.0026-23.2023.f3-1 Figure 3-1 In the table are results of 3 factors repeated-measures ANOVA (*ROI* × *Context* × *PBO-OT*) conducted in the Insula, ACC, NAcc, and the PHG. All results are Greenhouse-Geisser corrected. OT, oxytocin; PBO, placebo. Download Figure 3-1, DOCX file.

10.1523/JNEUROSCI.0026-23.2023.f3-2 Figure 3-2 Results of a 2 × 2 repeated-measures ANOVA (*Context* × *PBO-OT*) conducted for each of the 5 ROIs with above-threshold ISC, i.e., involved in stimulus processing. The insula and the PHG showed a significant main effect for *Context*. Results are Greenhouse-Geisser corrected. Abbreviations: ACC, anterior cingulate cortex; DMN, default mode network; NAcc, nucleus accumbens; OT, oxytocin; PBO, Placebo; PHG, parahippocampal gyrus;. **, *p* < .001. Download Figure 3-2, DOCX file.

10.1523/JNEUROSCI.0026-23.2023.f3-3 Figure 3-3 A 2 × 2 repeated-measures ANOVA (*Context* × *PBO-OT*) was performed to each of the 118 parcels involved in stimulus processing. The table presents 27 parcels that showed a significant main effect for *Context*, together with their anatomical location as defined by Neurosynth, F scores, *p* values, cluster sizes and cluster peak voxel. L, left; R, right. Download Figure 3-3, DOCX file.

10.1523/JNEUROSCI.0026-23.2023.f3-4 Figure 3-4 Statistical meta-analytic brain activation map of the term “social” across 1302 studies. The map was obtained through Neurosynth. RH, Right hemisphere; LH, left hemisphere. Download Figure 3-4, TIF file.

10.1523/JNEUROSCI.0026-23.2023.f3-5 Figure 3-5 A 2 × 2 repeated-measures ANOVA (*Context* × *PBO-OT*) yielded a significant *Context* effect in 15 parcels in addition to PCN regions, including the motor, somatosensory, visual, and orbitofrontal cortices. Their ISC and SE under the *Alone* (in blue) and the *Social* (in yellow) contexts are presented in the figure. Results are FDR-corrected. OFC, Orbitofrontal cortex. Download Figure 3-5, TIF file.

The second hypothesis was also supported by data-driven analyses, which identified PCN regions and “social brain” areas with higher ISC in the social context versus the Alone context. A 2 × 2 repeated-measures ANOVA (*Context* × *PBO-OT*) was performed for each of the 118 parcels involved in stimulus processing. In 27 parcels, a significant *Context* FDR-corrected effect for was found, with the *Social* context producing higher ISC compared with the *Alone* context (Fig. 3*B*). The ACC, insula, superior temporal and parahippocampal cortex, orbitofrontal cortex (OFC), PFC, areas in the striatum, motor, and premotor cortices were among those parcels. Results in the insula and PHG are consistent in both research approaches.

Regarding the third preregistered hypothesis which proposed enhanced ISC under OT compared with PBO, we found no significant differences between PBO and OT, neither in data-, nor in theory-driven analyses.

### Cross-brain synchrony tracks moment-by-moment variations in behavioral synchrony

Finally, we examined whether moments of behavioral synchrony induced greater neural synchrony between subjects, compared with nonsynchronous moments. This was tested in brain areas that were involved only in the *Social* context processing under PBO or OT: 2 ROIs (ACC and insula) and 42 parcels.

In the preregistered ACC, moments of greater mother–infant behavioral synchrony were associated with greater cross-brains synchrony between participants (*r_p_* = 0.445, *p* = 0.005) (Fig. 4*A*).

10.1523/JNEUROSCI.0026-23.2023.f4-1 Figure 4-1 Non-significant brain–behavior Pearson's correlation results in ROIs. NAcc, Nucleus accumbens; PHG, parahippocampal gyrus; DMN, default mode network. Download Figure 4-1, DOCX file.

10.1523/JNEUROSCI.0026-23.2023.f4-2 Figure 4-2 Brain–behavior correlations results of data-driven analyses. The figure depicts positive significant results of Pearson's correlations (not FDR-corrected) between neural similarity (purple line) and mother–infant behavioral synchrony (yellow line) in the *Free-play* video. Findings in the motor, primary-motor, and somatosensory cortices, left and right OFC and cerebellum are presented. OFC, Orbitofrontal cortex; R, right; L, left. Download Figure 4-2, TIF file.

Similar findings were revealed in 11 parcels, in which more synchronous behavior yielded more synchronous brain activity among participants (Fig. 4*B*). This was found in areas of the “social brain” (i.e., OFC) and the PCN: right ACC (rp = 0.42, *p* = 0.009) left insula (rp = 0.41, *p* = 0.01), right ventrolateral PFC (rp = 0.38, *p* = 0.019) under PBO and OFC (right: rp = 0.41, *p* = 0.01; left: rp = 0.35, *p* = 0.03), right dorsolateral PFC (DLPFC) (rp = 0.36, *p* = 0.02) and the right insula (rp = 0.35, *p* = 0.03), as well as in regions within the motor cortex (rp = 0.43, *p* = 0.007; rp = 0.35, *p* = 0.03), somatosensory cortex (rp = 0.35, *p* = 0.03), and in the cerebellum (rp = 0.38, *p* = 0.02). However, data-driven results did not remain significant after FDR correction because of multiple comparisons.

## Discussion

The mother–infant bond provides the main context for survival, growth, and safety of the young and reminders of the primary attachment, whether stimuli of one's own child or those of a standard infant (Ranote et al., 2004; Kringelbach et al., 2008; Noriuchi et al., 2008; Mascaro et al., 2014), trigger a powerful response in the brains of human adults (Swain, 2011) as they do in other mammals (Vom Saal, 1985; Lonstein and De Vries, 2000; Novakov and Fleming, 2005; Olazábal and Young, 2006). While research has pinpointed brain regions that activate in response to attachment reminders (Swain, 2011; Swain et al., 2014; Rigo et al., 2019), our results are the first to show widespread cross-brains synchronization to the presentation of dynamic mother–infant stimuli. We found that large portions of the brain, indeed 44% of the parcels measured, activate in tandem to the presentation of attachment reminders, indicating that bonding-related cues reduce variability among individual brains and enhance their synchronous, uniform response. Areas of cross-brains concordance were widespread, from lower-level visual, auditory, and somatosensory cortices, to subcortical limbic and paralimbic regions, to cortical prefrontal and orbitofrontal areas. It thus appears that representations of the primary attachment not only elicit substantial neural response but also generate significant uniformity among multiple brains.

Our findings indicate that, while cross-brains similarity in largely distributed networks follows any reminder of the primary attachment, the *social* mother–infant context elicits significantly greater neural concordance. Indeed, 27 parcels (23% of the “synchronized parcels”), including regions of the PCN as well as sensorimotor, visual, auditory, and higher-level regions, activated in tandem to the presentation of the mother–infant *social* contexts, not to the *alone* contexts. In addition to most areas of the PCN, results also show cross-brains correlations in areas of sensory processing and integration, mentalization, association, and higher-order valuation. Importantly, our results show, for the first time, that moment-by-moment variability in the magnitude of cross-brains synchronization tracks online fluctuations in the level of mother–infant behavioral synchrony in the presented video and pinpoint the ACC (in the top-down analysis) and insula (in the bottom-up analysis), areas that showed greater activation to own attachment stimuli in the same sample, in this cross-brains- behavior linkage. These findings are the first to highlight parallels between a mother's brain responsivity to her exclusive attachment and her cross-brains synchronization to generic attachment reminders.

Mother–infant synchrony is a core human-specific behavioral mechanism that provides a framework for the online synchronization of physiological (Feldman et al., 2011) and neural (Endevelt-Shapira et al., 2021) responses between mother and child during social interactions. Longitudinal studies show that mother–infant synchrony predicts children's social-emotional competencies in group settings and shape their neural response to social and attachment stimuli in adolescence and young adulthood (Feldman, 2016, 2017, 2021b; Yaniv et al., 2021). The nonverbal behavioral building-blocks of mother–infant synchrony (gaze, affect, touch, and vocalizations) characterize interactions between all mothers and infants in our species and, across cultures, assume a repetitive-rhythmic, highly coordinated expression. It has been suggested that, through mechanisms of *biobehavioral synchrony* that coordinate online physiological processes and social signals, mothers usher infants into the social world (Feldman, 2021a). Our findings raise the possibility that these moments of synchrony not only strengthen the mother–infant attachment but also create a potential for enhanced uniformity among multiple brains, thereby cementing a neural template for the consolidation of individuals into social groups.

Our findings contribute to the literature on cross-brains synchronization by demonstrating a widespread cross-brain concordance to nonverbal stimuli that present no overt narrative. Most previous studies on cross-brains concordance focused on the unfolding of stories with a clear narrative (Hasson et al., 2008; Wilson et al., 2008; Nguyen et al., 2019; Redcay and Moraczewski, 2020) and only a few presented stimuli with no verbal narrative, such as a dance performance, musical piece, or action observation (Herbec et al., 2015; Kostorz et al., 2020; Sachs et al., 2020). Stimuli of the mother–infant bond are fundamental and can be understood universally without the need for words. The widespread neural concordance found here suggests that human cross-brain similarity does not rely solely on higher-order mechanisms of shared mentalization, language, and cognition but also include key experiences of human sociality. Our study indicates that in the context of the mother–infant attachment, there is no need for a complex and elaborate narrative to synchronize the perceivers' brain responses across widely-distributed networks.

Attachment reminders triggered substantial cross-brains synchronization across the neuroaxis. Regions displaying cross-brains concordance to both *Social* and *Alone* attachment reminders spanned from the occipital cortex to the PFC, through temporal, limbic, and paralimbic regions. This is consistent with studies that measured cross-brains synchronization to naturalistic videos and showed high and stable ISC across extensive brain areas; from occipital regions, including the visual cortex, fusiform gyrus, and the precuneus, to frontal regions, including the OFC and temporal regions, and paralimbic regions, including the insula (Kauppi et al., 2010; Gao et al., 2020). Here, the broadband interindividual correlated response included regions of the caregiving network as well as other subcortical regions. This points to the universal nature of the mother–infant bond and its evolutionary significance and suggests that attachment reminders not only recruit substantial resources but also glue mothers' brains into greater uniformity. Our stimuli comprising daily mother–infant ecological contexts were highly relevant to the participants, who were at the stage of forming a bond with their infant and allocating their physical, mental, and emotional energies to this survival-related task (Tronick, 1989; Feldman, 2020). Our widespread results are also consistent with studies that showed ISC increase when individuals have similar interests, share a psychological perspective, or empathize with the stimuli (Lahnakoski et al., 2014; Borja Jimenez et al., 2020; Hyon et al., 2020). Possibly, the correlated activations of the PCN in postpartum mothers to generic mother–infant stimuli stem from their empathy with the mother in the video, their shared mental focus, and their ability to take her perspective, which are reflected in the enhanced neural resemblance.

Still, representations of mother and infant together (*Social*) elicited significantly greater neural concordance compared with stimuli of mother or infant alone (*Alone*). Cross-brains synchronization only to the *social* contexts emerged in the insula, ACC, PHG, STG, PFC, and striatum. The insula and the ACC are selectively responsive to self-related stimuli (Benuzzi et al., 2018; Esménio et al., 2019; Ulmer-Yaniv et al., 2022) and in a previous study with the same cohort showed sensitivity to Self-Other distinctions with a greater response to self-related mother–infant stimuli (Shimon-Raz et al., 2021). Imaging studies that exposed parents to photographs or videos of an unfamiliar infant report activations in the PFC, insula, ACC, PHG, and amygdala (Bartels and Zeki, 2004; Leibenluft et al., 2004; Nitschke et al., 2004; Strathearn et al., 2009; Landi et al., 2011). Together, these findings suggest that infant stimuli have a discernible impact on the PCN, with specific regions exhibiting increased sensitivity and reactivity to social compared with alone stimuli as well as a greater response to stimuli related to one's own infant compared with unfamiliar infants. Activations in these regions to parents' own infant were found to correlate with the degree of their behavioral synchrony (Atzil et al., 2014; Abraham et al., 2017; Shimon-Raz et al., 2021; Ulmer-Yaniv et al., 2022). Of note, regions showing a main effect for the *Social* context also overlap with areas reported in a meta-analytic map of the term “social” that tested response to social processes across 1302 studies (Fig. 3*B*; Extended Data Fig. 3-4) (Yarkoni et al., 2011). Our findings are the first to show that these social areas not only activate to social stimuli but also exhibit greater convergence to attachment-related social stimuli. It appears that two-person representations of attachment generate lower variability of neural response across participants compared with single-person reminders. Notably, auditory, visual, and somatosensory low-level regions also showed greater synchrony to the *Social* context. This may relate to the multimodal nature of mother–infant interactions or from top-down circuitry based on the salience of these stimuli, but this hypothesis requires further research.

Both the top-down and bottom-up analyses demonstrated the involvement of the PCN in the convergent cross-brain processing of the stimuli. This highlights the centrality of the PCN from a new angle: the network that underpins human caregiving and activates to attachment-related cues in parents (Abraham et al., 2014; Atzil et al., 2014), children (Pratt et al., 2018; Ulmer-Yaniv et al., 2022), romantic couples (Acevedo et al., 2012; Scheele et al., 2013), and close friends (Platek and Kemp, 2009; Parkinson et al., 2018) also binds humans' brains into a convergent response and enhances neural uniformity. The PCN includes conserved subcortical areas implicated in mammalian maternal care that are connected via multiple ascending and descending projections to insular, temporal, and frontal regions (Rilling and Young, 2014; Feldman, 2015) to sustain human attachment. Our findings highlight the openness of this network to cross-brain processes in the presence of attachment-related cues, which may function to bind humans into social groups by leveling-out variability among brains.

The covariability of ISC with shifts in behavioral synchrony is among the novel findings of our study. We found that fluctuations in the activity of the preregistered ACC tracked moment-by-moment variations in mother–infant synchrony, presenting the first evidence that links cross-brain synchronization with fluctuations in behavioral synchrony of the presented stimulus. The ACC is densely connected with sensory, limbic, and paralimbic regions (Etkin et al., 2006, 2011), underpins sensation, affective behavior, and decision-making (Peterson et al., 1999; Bush et al., 2000; Pavlvlović et al., 2009), and supports self-relational processes (Northoff et al., 2006; Ulmer-Yaniv et al., 2022). The ACC also participates in social observational learning in animals and humans (Burgos-Robles et al., 2019), including observational fear learning in rodents (Allsop et al., 2018) and social decision-making in monkeys (Chang et al., 2013). Furthermore, the ACC has been shown to play a key role in brain–behavior linkage in the context of caregiving. For instance, the degree of mother–infant behavioral synchrony during free play correlated with her ACC response to the presentation of synchronous versus asynchronous generic mother–infant videos (Atzil et al., 2014). Mothers' ACC response to her own infant video, compared with unfamiliar infant, correlated with her behavioral synchrony observed in the home environment (Abraham et al., 2014). Moreover, young adults viewing videos of their own interactions with their mother across infancy, childhood, and adulthood, compared with unfamiliar videos, not only showed greater ACC response to self-videos but also increased functional connectivity between ACC and insula in response to attachment reminders (Ulmer-Yaniv et al., 2022), highlighting the ACC–insula interface in context of attachment (Dosenbach et al., 2007; Medford and Critchley, 2010). Here we add to the ACC's role in the consolidation of attachment representations, also the integrative function of synchronizing activity across multiple brains in a manner that binds all brains to the presented attachment reminders.

Several studies examined neural synchrony between mother and infant or an adult and infant during live social interactions using hyperscanning methods (Piazza et al., 2021). Interestingly, these studies show mother–infant neural synchrony in areas that were found here to exhibit cross-brain ISC, including the STS (Endevelt-Shapira et al., 2021) and PFC (Piazza et al., 2020; Nguyen et al., 2021). Synchronous mother–infant interactions were found to increase mother–child frontal-frontal and temporal-temporal synchrony (Endevelt-Shapira and Feldman, 2023). These findings point to the role of the PCN in providing the neural template for behavioral synchrony (Atzil et al., 2011; Abraham et al., 2014), brain-to-brain synchrony during live social moments (Endevelt-Shapira and Feldman, 2023), as well as cross-brains synchronization in response to attachment reminders.

Finally, while our first two preregistered hypotheses were supported by the findings, the third hypothesis, which postulated greater cross-brains synchrony under OT, was not. OT has been repeatedly linked with parental caregiving behaviors (Olazábal and Young, 2006; Levine et al., 2007; Galbally et al., 2011) and OT administration modified activations in the PCN to attachment stimuli (Riem et al., 2011a; Owen et al., 2013; Wu et al., 2023). However, unlike levels of activation, no differences emerged in cross-brain correlations to attachment reminders under OT versus PBO in the social and alone contexts. Prior studies showed that OT modulated functional connectivity within and between networks, including the DMN and salience network (Jiang et al., 2021; Zheng et al., 2021; Wu et al., 2023), but cross-brains synchronization to attachment stimuli has not been studied. Possibly, attachment-related stimuli elicit such broadband cross-brains resemblance in postpartum mothers that this may have created a ceiling effect. Overall, our results are consistent with the consensus in the field that OT effects are person-, time-, and context-sensitive (Bartz et al., 2010).

Limitations of the study mainly concern the specificity of our sample. As this is the first study of cross-brains synchronization to attachment stimuli, we chose to test mothers for whom such stimuli are the most relevant, but future studies are needed to examine fathers and nonparents. We believe that representations of the mother–infant attachment would function to create neural uniformity in all adult members of our species, but such hypothesis requires further research. Further research is also needed to assess whether psychopathologies involving social dysfunction, such as autism or depression, which have been associated with diminished cross-brain synchronization to a variety of stimuli (Salmi et al., 2013; Komulainen et al., 2021), also exhibit attenuated ISC to infant stimuli, and particularly relevant in this context are cases of postpartum depression. It would be of interest to see cross-brains synchronization to father–infant attachment and whether and how such synchrony differs from the mother–infant attachment. Finally, it is important to study cross-brains neural concordance to negatively valance attachment cues, such as infant cry or distress, and whether they trigger similar cross-brains resemblance as do the positive attachment reminders. Finally, as we preferred using real-life naturalistic stimuli, our stimuli are not fully controlled for low-level properties, a condition that is generally an issue in ecological brain research.

To our knowledge, this is the first study to show neural synchronization across multiple brains in response to attachment-related stimuli. We showed, using both theory-driven and data-driven analytic approaches, a widespread cross-brains synchronization to attachment reminders that was even greater in response to mother–infant social cues. Cross-person correlations in the ACC dynamically tracked moment-by-moment variations in mother–infant behavioral synchrony, suggesting that moments of coordinated behavior between mother and child trigger cross-brains uniformity. Together, our findings highlight the primary attachment as a core survival-related phenomenon that recruits substantial neural resources, and suggest that attachment bonds may function to cement a neural template for the consolidation of humans into social groups.
