# Supplementary material for: Attachment Reminders Trigger Widespread Synchrony across Multiple Brains
Source: J Neurosci. 2023 Oct 25;43(43):7213–25. doi: 10.1523/JNEUROSCI.0026-23.2023 (PMC10601370; doi:10.1523/JNEUROSCI.0026-23.2023)
Supplement: Figure 1-1 — 2 × 2 Bayesian repeated-measures ANOVA results (Infant/Mother alone video× PBO-OT) demonstrated evidence for the lack of difference between the 2 Alone context videos ISCs, between PBO and OT and the lack of interaction effect between them, through the PCN. Abbreviations: OT, Oxytocin; PBO, Placebo; PCN, Parental Caregiving Network. Download Figure 1-1, DOCX file. [file ns-JN-RM-0026-23-s01.docx]

|  | $\boldsymbol{B}\boldsymbol{F}_{\boldsymbol{10}}$ | $\boldsymbol{B}\boldsymbol{F}_{\boldsymbol{incl}}$ |
| --- | --- | --- |
| *Mother alone / Infant alone* main effect | 0.24 | 0.16 |
| *PBO-OT* main effect | 0.22 | 0.18 |
| *Mother alone / Infant alone × PBO-OT* interaction | 0.05 | 0.07 |

**Figure 1-1**. 2*×*2 Bayesian repeated measures ANOVA results (*Infant*/*Mother alone* video *×* *PBO-OT*) in the PCN.
