# Supplementary material for: Attachment Reminders Trigger Widespread Synchrony across Multiple Brains
Source: J Neurosci. 2023 Oct 25;43(43):7213–25. doi: 10.1523/JNEUROSCI.0026-23.2023 (PMC10601370; doi:10.1523/JNEUROSCI.0026-23.2023)
Supplement: Figure 1-2 — 2 × 2 Bayesian repeated-measures ANOVA results (Free-play/Breastfeeding video × PBO/OT) revealed the lack of difference between the 2 Social context videos ISCs between PBO and OT and the lack of interaction effect between them, through the PCN. Abbreviations: OT, Oxytocin; PBO, Placebo; PCN, Parental Caregiving Network. Download Figure 1-2, DOCX file. [file ns-JN-RM-0026-23-s02.docx]

|  | $\boldsymbol{B}\boldsymbol{F}_{\boldsymbol{10}}$ | $\boldsymbol{B}\boldsymbol{F}_{\boldsymbol{incl}}$ |
| --- | --- | --- |
| *Breastfeeding / Free-play* main effect | 0.34 | 0.79 |
| *PBO-OT* main effect | 0.24 | 0.73 |
| *Breastfeeding / Free-play × PBO-OT* interaction | 0.08 | 0.98 |

**Figure 1-2**. 2*×*2 Bayesian repeated measures ANOVA results (*Free-play*/*Breastfeeding* video *×* *PBO-OT*) in the PCN.
