# Supplementary figures and images for: Attachment Reminders Trigger Widespread Synchrony across Multiple Brains
Source: J Neurosci. 2023 Oct 25;43(43):7213–25. doi: 10.1523/JNEUROSCI.0026-23.2023 (PMC10601370; doi:10.1523/JNEUROSCI.0026-23.2023)

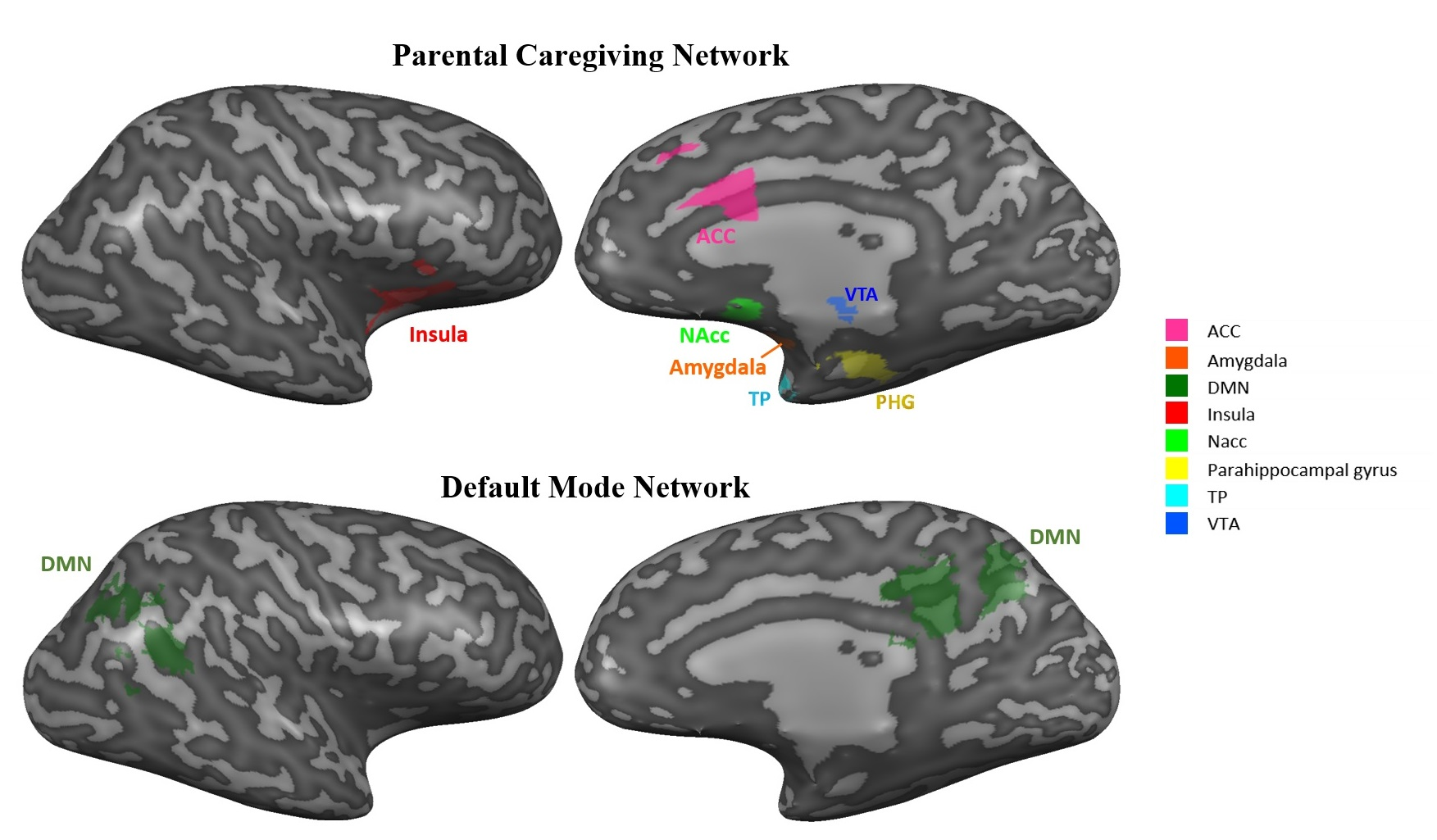

Supplement: Figure 1-3 — Eight preregistered ROIs, including the ACC, amygdala, insula, NAcc, PHG, TP, VTA in the PCN (upper), and the DMN (footer). Abbreviations: ACC, anterior cingulate cortex; DMN, default mode network; NAcc, nucleus accumbens, PCN, parental caregiving network; PHG, parahippocampal gyrus; TP, temporal pole; VTA, ventral tegmental area. Download Figure 1-3, TIF file. [file ns-JN-RM-0026-23-s03.tif]

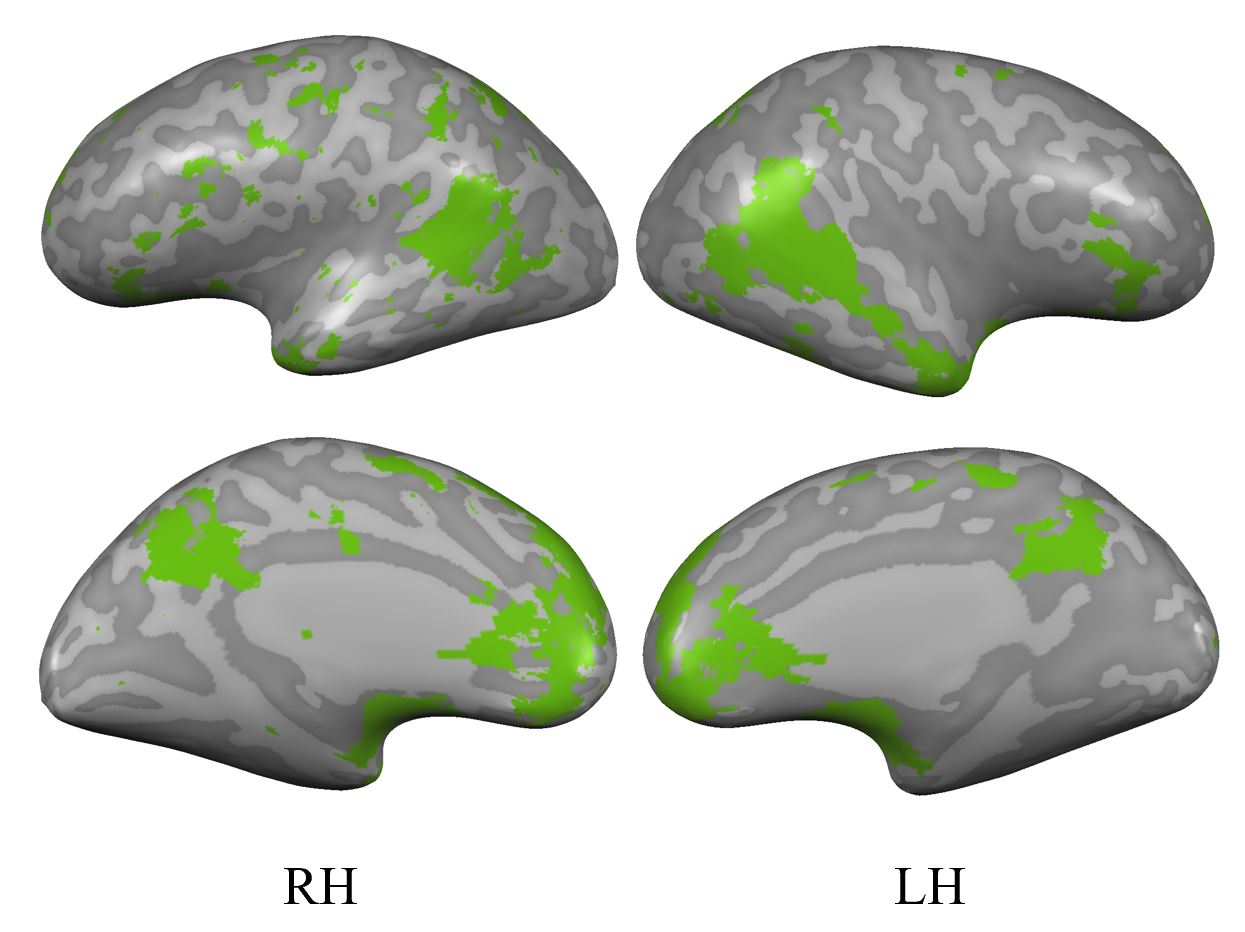

Supplement: Figure 3-4 — Statistical meta-analytic brain activation map of the term “social” across 1302 studies. The map was obtained through Neurosynth. RH, Right hemisphere; LH, left hemisphere. Download Figure 3-4, TIF file. [file ns-JN-RM-0026-23-s11.tif]

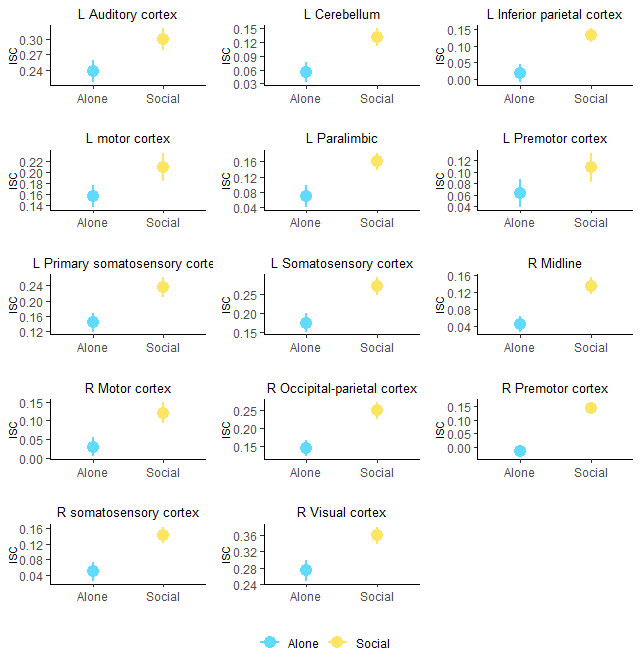

Supplement: Figure 3-5 — A 2 × 2 repeated-measures ANOVA (Context × PBO-OT) yielded a significant Context effect in 15 parcels in addition to PCN regions, including the motor, somatosensory, visual, and orbitofrontal cortices. Their ISC and SE under the Alone (in blue) and the Social (in yellow) contexts are presented in the figure. Results are FDR-corrected. OFC, Orbitofrontal cortex. Download Figure 3-5, TIF file. [file ns-JN-RM-0026-23-s12.tif]

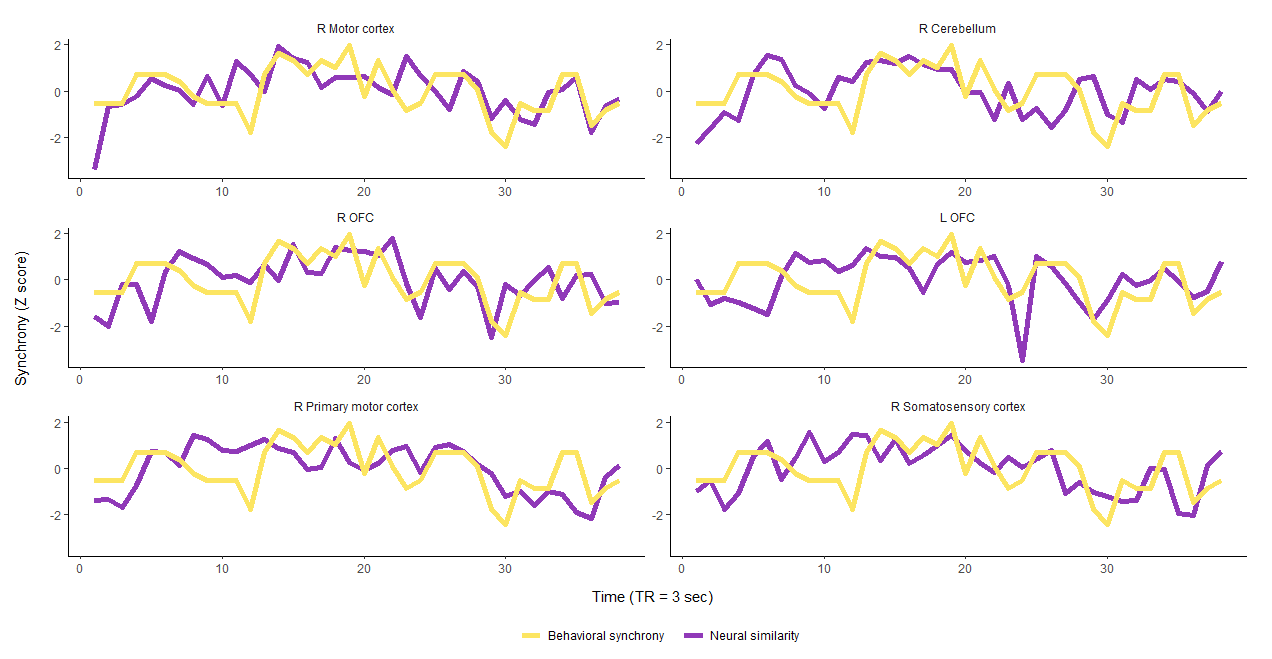

Supplement: Figure 4-2 — Brain–behavior correlations results of data-driven analyses. The figure depicts positive significant results of Pearson's correlations (not FDR-corrected) between neural similarity (purple line) and mother–infant behavioral synchrony (yellow line) in the Free-play video. Findings in the motor, primary-motor, and somatosensory cortices, left and right OFC and cerebellum are presented. OFC, Orbitofrontal cortex; R, right; L, left. Download Figure 4-2, TIF file. [file ns-JN-RM-0026-23-s14.tif]
