# Supplementary material for: Attachment Reminders Trigger Widespread Synchrony across Multiple Brains
Source: J Neurosci. 2023 Oct 25;43(43):7213–25. doi: 10.1523/JNEUROSCI.0026-23.2023 (PMC10601370; doi:10.1523/JNEUROSCI.0026-23.2023)
Supplement: Figure 1-4 — Behavioral coding of mother–infant synchronization during Free-play interaction. Behavior was coded over 3 non-verbal parameters: gaze, affect, and vocalization. The synchronization level indicates the degree of compatibility between the mother and the baby, with a higher score indicating a higher level of behavioral synchronization. Download Figure 1-4, DOCX file. [file ns-JN-RM-0026-23-s04.docx]

|  | Infant | | | Mother | | |
| --- | --- | --- | --- | --- | --- | --- |
| behavioral synchrony level | Vocalization | Gaze | Affect | Vocalization | Gaze | Affect |
| **1** | no vocalization | aversion | neutral | Motherese | to infant's face | neutral |
| **2** | no vocalization | to environment/object | neutral | Motherese | to infant's face | neutral |
| **3** | no vocalization | to mother | neutral | Motherese | to infant's face | neutral |
| **4** | no vocalization | to environment/object | positive (smile) | Motherese | to infant's body | neutral |
| **5** | no vocalization | to mother | neutral | Motherese | to infant's face | positive (smile) |
| **6** | no vocalization | to mother | positive (smile) | Motherese | to infant's body | neutral |
| **7** | no vocalization | to mother | positive (smile) | Motherese | to infant's face | neutral |
| **8** | cooing | to mother | positive (smile) | Motherese | to infant's face | neutral |
| **9** | no vocalization | to mother | positive (smile) | Motherese | to infant's body | positive (smile) |
| **10** | no vocalization | to environment/object | positive (smile) | Motherese | to infant's face | positive (smile) |
| **11** | no vocalization | to mother | positive (smile) | Motherese | to infant's face | positive (smile) |
| **12** | cooing | to environment/object | positive (smile) | Motherese | to infant's body | positive (smile) |
| **13** | cooing | to mother | positive (smile) | Motherese | to infant's body | positive (smile) |
| **14** | cooing | to mother | positive (smile) | Motherese | to infant's face | positive (smile) |
| **15** | laugh | to mother | positive (smile) | Motherese | to infant's face | positive (smile) |

**Figure 1-4**. Behavioral coding of mother-infant synchronization during *Free-play* interaction.
