# Supplementary material for: Attachment Reminders Trigger Widespread Synchrony across Multiple Brains
Source: J Neurosci. 2023 Oct 25;43(43):7213–25. doi: 10.1523/JNEUROSCI.0026-23.2023 (PMC10601370; doi:10.1523/JNEUROSCI.0026-23.2023)
Supplement: Figure 2-1 — ISC scores of the parcels involved in stimulus processing in one condition or more. Only above-threshold values are displayed. Download Figure 2-1, DOCX file. [file ns-JN-RM-0026-23-s05.docx]

**Figure 2-1.** 118 parcels involved in stimulus processing ISCs.

| Parcel number | Anatomical area | ISC | | | |
| --- | --- | --- | --- | --- | --- |
|  |  | ***PBO Alone*** | ***PBO Social*** | ***OT Alone*** | ***OT Social*** |
| Exclusive to *Social* context | |  |  |  |  |
| 8 | Prefrontal ventrolateral lateral parietal | – | 0.152 | – | – |
| 16 | Insula | – | 0.169 | – | – |
| 33 | Motor cortex | – | 0.157 | – | – |
| 96 | Hippocampal gyrus medial temporal | – | 0.157 | – | – |
| 100 | Cerebellum | – | 0.153 | – | – |
| 123 | Striatum ventral striatum | – | 0.161 | – | – |
| 125 | Striatum ventral striatum | – | 0.165 | – | – |
| 147 | Dorsolateral PFC | – | 0.174 | – | – |
| 150 | ACC | – | 0.146 | – | – |
| 162 | Motor cortex | – | 0.181 | – | – |
| 165 | Premotor cortex | – | 0.162 | – | – |
| 188 | Temporal pole | – | 0.190 | – | – |
| 225 | Precuneus | – | 0.156 | – | – |
| 4 | Orbitofrontal cortex | – | – | – | 0.182 |
| 14 | Dorsolateral PFC | – | – | – | 0.147 |
| 19 | Frontoparietal | – | – | – | 0.199 |
| 30 | Prefrontal parietal | – | – | – | 0.152 |
| 32 | Premotor cortex | – | – | – | 0.193 |
| 39 | Somatosensory cortex | – | – | – | 0.182 |
| 89 | Midline | – | – | – | 0.185 |
| 91 | precuneus | – | – | – | 0.199 |
| 137 | Orbitofrontal cortex | – | – | – | 0.166 |
| Parcel number | **Anatomical area** | **ISC** | | | |
|  |  | ***PBO Alone*** | ***PBO Social*** | ***OT Alone*** | ***OT Social*** |
| 139 | Orbitofrontal cortex | – | – | – | 0.149 |
| 142 | Prefrontal cortex | – | – | – | 0.147 |
| 144 | Prefrontal cortex | – | – | – | 0.169 |
| 166 | Premotor | – | – | – | 0.221 |
| 169 | Putamen, striatum | – | – | – | 0.165 |
| 230 | Parahippocampal cortex | – | – | – | 0.155 |
| 240 | Cerebellum | – | – | – | 0.203 |
| 246 | Cerebellum | – | – | – | 0.173 |
| 259 | Striatum accumbens | – | – | – | 0.146 |
| 27 | Premotor | – | 0.168 | – | 0.15 |
| 37 | Insula | – | 0.154 | – | 0.192 |
| 53 | Temporal pole, superior temporal | – | 0.198 | – | 0.175 |
| 143 | Prefrontal cortex | – | 0.184 | – | 0.152 |
| 155 | Anterior insula | – | 0.143 | – | 0.149 |
| 170 | Insula | – | 0.162 | – | 0.185 |
| 173 | Posterior insula | – | 0.170 | – | 0.146 |
| 184 | Parietal ipl | – | 0.169 | – | 0.164 |
| 192 | Superior temporal | – | 0.250 | – | 0.194 |
| 217 | Paralimbic | – | 0.206 | – | 0.163 |
| Exclusive to *Alone* context | |  |  |  |  |
| 243 | Cerebellum | – | – | 0.153 | – |
| Involved in *Alone* and *Social* contexts | |  |  |  |  |
| 242 | Cerebellum | 0.161 | 0.152 | – | – |
| 44 | Superior parietal | – | – | 0.160 | 0.171 |

| Parcel number | Anatomical area | ISC | | | |
| --- | --- | --- | --- | --- | --- |
|  |  | ***PBO Alone*** | ***PBO Social*** | ***OT Alone*** | ***OT Social*** |
| 86 | Posterior cingulate precuneus posterior | – | – | 0.160 | 0.197 |
| 177 | Intraparietal sulcus | – | – | 0.142 | 0.199 |
| 178 | Parietal | – | – | 0.163 | 0.165 |
| 183 | Temporoparietal junction | – | – | 0.138 | 0.145 |
| 208 | Occipital visual | – | – | 0.190 | 0.172 |
| 224 | Parietal junction, posterior cingulate | – | – | 0.164 | 0.159 |
| 226 | Sensorimotor | – | – | 0.152 | 0.196 |
| 22 | Dorsolateral PFC | 0.217 | 0.175 | 0.174 | 0.236 |
| 26 | Motor cortex sensorimotor | 0.156 | 0.155 | 0.150 | – |
| 31 | Temporal parietal | 0.158 | 0.146 | 0.162 | 0.195 |
| 38 | Premotor | 0.247 | 0.241 | 0.231 | 0.257 |
| 41 | Premotor | 0.147 | 0.198 | 0.155 | 0.249 |
| 42 | Precuneus | 0.158 | 0.145 | 0.187 | 0.185 |
| 43 | Parietal cortex | 0.181 | 0.172 | – | 0.231 |
| 45 | Somatosensory | 0.143 | 0.168 | 0.186 | 0.235 |
| 46 | Somatosensory | 0.301 | 0.344 | 0.287 | 0.341 |
| 47 | Inferior parietal | 0.138 | 0.151 | – | 0.150 |
| 48 | Default mode** | 0.197 | 0.166 | 0.157 | 0.161 |
| 49 | Medial temporal | 0.252 | 0.245 | 0.284 | 0.328 |
| 50 | Temporal sulcus | 0.302 | 0.268 | 0.279 | 0.279 |
| 54 | Temporal sulcus superior temporal | 0.211 | 0.211 | 0.312 | 0.196 |
| 61 | primary auditory | 0.242 | 0.285 | 0.224 | 0.256 |
| 62 | posterior insula | 0.179 | 0.280 | 0.218 | 0.270 |
| 63 | Superior temporal | 0.241 | 0.340 | 0.225 | 0.227 |

| Parcel number | Anatomical area | ISC | | | |
| --- | --- | --- | --- | --- | --- |
|  |  | ***PBO Alone*** | ***PBO Social*** | ***OT Alone*** | ***OT Social*** |
| 64 | Lateral temporal | 0.209 | 0.214 | 0.158 | 0.176 |
| 65 | Temporal sulcus | 0.352 | 0.297 | 0.294 | 0.283 |
| 66 | Fusiform | 0.247 | 0.240 | 0.203 | 0.245 |
| 67 | Fusiform | 0.254 | 0.213 | 0.236 | 0.251 |
| 68 | Parahippocampal | 0.223 | 0.238 | 0.276 | 0.282 |
| 69 | Visual cortex | 0.224 | 0.209 | 0.197 | 0.270 |
| 71 | Fusiform | 0.189 | 0.191 | 0.179 | 0.216 |
| 72 | Visual fusiform | 0.194 | 0.187 | 0.230 | 0.240 |
| 73 | Visual cortex occipital | 0.256 | 0.341 | 0.274 | 0.403 |
| 74 | Visual cortex occipital | 0.378 | 0.368 | 0.271 | 0.432 |
| 75 | Occipital parietal | – | 0.220 | 0.161 | 0.264 |
| 76 | Visual occipital | 0.220 | 0.246 | 0.267 | 0.244 |
| 77 | Cuneus | 0.222 | 0.181 | 0.220 | 0.230 |
| 78 | Occipital gyrus | 0.340 | 0.362 | 0.307 | 0.414 |
| 79 | Visual cortex | 0.230 | 0.179 | 0.282 | 0.215 |
| 80 | Occipital cuneus | 0.221 | 0.198 | 0.277 | 0.214 |
| 81 | Inferior occipital | 0.306 | 0.245 | 0.278 | 0.302 |
| 82 | Parietal network visual | 0.216 | 0.152 | 0.221 | 0.164 |
| 87 | Accumbens | 0.148 | – | – | 0.155 |
| 90 | Precuneus | 0.147 | 0.153 | 0.141 | 0.170 |
| 95 | Hippocampus | 0.141 | – | – | 0.196 |
| 98 | Hippocampus | – | 0.148 | 0.155 | – |
| 163 | Somatosensory cortices | – | 0.266 | 0.175 | 0.227 |
| 166 | Premotor | 0.211 | 0.208 | 0.190 | 0.221 |

| Parcel number | Anatomical area | ISC | | | |
| --- | --- | --- | --- | --- | --- |
|  |  | ***PBO Alone*** | ***PBO Social*** | ***OT Alone*** | ***OT Social*** |
| 171 | Motor cortex | 0.147 | 0.237 | 0.141 | 0.232 |
| 175 | Premotor | 0.203 | 0.195 | 0.182 | 0.253 |
| 176 | Precuneus | 0.158 | 0.162 | 0.155 | 0.183 |
| 179 | Intraparietal premotor | 0.217 | 0.253 | 0.204 | 0.273 |
| 180 | Auditory cortex | 0.279 | 0.368 | 0.295 | 0.354 |
| 181 | Somatosensory cortex | 0.155 | 0.278 | 0.179 | 0.293 |
| 182 | Angular gyrus | – | 0.169 | 0.139 | – |
| 191 | Auditory cortex | 0.206 | 0.352 | 0.242 | 0.297 |
| 197 | Auditory cortex superior temporal | 0.158 | 0.273 | – | 0.161 |
| 198 | Parahippocampal | 0.153 | – | 0.220 | 0.202 |
| 200 | Fusiform | 0.184 | 0.188 | 0.227 | 0.204 |
| 203 | Visual motion default mode | 0.230 | 0.185 | 0.284 | 0.225 |
| 204 | Occipital cortex | 0.216 | 0.277 | 0.270 | 0.299 |
| 205 | Parahippocampal cortex | 0.163 | 0.149 | 0.139 | 0.194 |
| 206 | Visual occipital fusiform | 0.254 | 0.199 | 0.249 | 0.238 |
| 207 | Fusiform | 0.159 | 0.145 | 0.219 | 0.150 |
| 209 | Visual | 0.294 | 0.350 | 0.279 | 0.364 |
| 210 | Visual occipital fusiform | 0.289 | 0.273 | 0.309 | 0.286 |
| 211 | Early visual | 0.187 | 0.180 | 0.231 | 0.219 |
| 212 | Visual occipital | 0.227 | 0.207 | 0.253 | 0.272 |
| 213 | Visual | 0.159 | 0.162 | 0.208 | 0.180 |
| 214 | Inferior occipital | 0.264 | 0.242 | 0.252 | 0.298 |
| 215 | Cuneus | 0.218 | 0.175 | 0.236 | 0.185 |
| 216 | Early visual | 0.174 | 0.192 | 0.158 | 0.197 |
| 247 | Cerebellum | 0.167 | – | – | 0.144 |
