# Supplementary material for: Attachment Reminders Trigger Widespread Synchrony across Multiple Brains
Source: J Neurosci. 2023 Oct 25;43(43):7213–25. doi: 10.1523/JNEUROSCI.0026-23.2023 (PMC10601370; doi:10.1523/JNEUROSCI.0026-23.2023)
Supplement: Figure 2-2 — ISC scores and p values of the predefined ROIs, and threshold values for each condition in the theory-driven analyses. Results are FDR-corrected. Abbreviations: OT, oxytocin; PBO, Placebo; *, ROIs with above-threshold ISC under at least one condition. Download Figure 2-2, DOCX file. [file ns-JN-RM-0026-23-s06.docx]

**Figure 2-2.** ISC threshold values for theory-driven analyses (ROIs).

| **ISC**  **(*p* value)** | **Threshold** | **TP** | **ACC*** | **Insula*** | **PHG*** | **Amygdala** | **NAcc*** | **VTA** | **DMN*** |
| --- | --- | --- | --- | --- | --- | --- | --- | --- | --- |
| *PBO Alone* | 0.12 | 0.0187  (0.311) | 0.0556  (0.053) | 0.0607  (0.027) | 0.0182  (0.235) | 0.0870  (0.002) | 0.1688  (0.001) | -0.0145  (0.659) | 0.1494  (0.001) |
| *PBO Social* | 0.13 | 0.0730  (0.022) | 0.1079  (0.002) | 0.1308  (0.001) | 0.1394  (0.001) | 0.0343  (0.128) | 0.1340  (0.001) | -0.0179  (0.675) | 0.1453  (0.001) |
| *OT Alone* | 0.12 | -0.0251  (0.795) | 0.1031  (0.004) | 0.0726  (0.008) | 0.0228  (0.267) | 0.0075  (0.716) | 0.1237  (0.001) | 0.0693  (0.005) | 0.0726  (0.001) |
| *OT Social* | 0.11 | 0.0860  (0.007) | 0.1656  (0.001) | 0.1665  (0.001) | 0.1074  (0.001) | 0.0296  (0.102) | 0.1606  (0.001) | 0.0481  (0.965) | 0.1665  (0.001) |
