# Supplementary material for: Attachment Reminders Trigger Widespread Synchrony across Multiple Brains
Source: J Neurosci. 2023 Oct 25;43(43):7213–25. doi: 10.1523/JNEUROSCI.0026-23.2023 (PMC10601370; doi:10.1523/JNEUROSCI.0026-23.2023)
Supplement: Figure 2-3 — ISC threshold values for each condition in the data-driven analyses (parcels). Download Figure 2-3, DOCX file. [file ns-JN-RM-0026-23-s07.docx]

| **Condition** | **ISC** |
| --- | --- |
| *PBO Alone* | 0.1379 |
| *PBO Social* | 0.1426 |
| *OT Alone* | 0.1368 |
| *OT Social* | 0.1413 |

**Figure 2-3.** ISC threshold values for data-driven analyses (parcels).
