# Supplementary material for: Attachment Reminders Trigger Widespread Synchrony across Multiple Brains
Source: J Neurosci. 2023 Oct 25;43(43):7213–25. doi: 10.1523/JNEUROSCI.0026-23.2023 (PMC10601370; doi:10.1523/JNEUROSCI.0026-23.2023)
Supplement: Figure 3-1 — In the table are results of 3 factors repeated-measures ANOVA (ROI × Context × PBO-OT) conducted in the Insula, ACC, NAcc, and the PHG. All results are Greenhouse-Geisser corrected. OT, oxytocin; PBO, placebo. Download Figure 3-1, DOCX file. [file ns-JN-RM-0026-23-s08.docx]

**Figure 3-1.** 4×2×2 repeated measures ANOVA (*ROI* × *Context* × *PBO-OT*) effects on ISC in PCN ROIs.

|  | df | F | *p* | Eta^2^ |
| --- | --- | --- | --- | --- |
| ROI main effect | 2.53, 58.40 | 5.21 | 0.003 | 0.185 |
| *Context* main effect | 1,23 | 10.96 | 0.003 | 0.323 |
| *PBO-OT*  main effect | 1,23 | 0.28 | 0.604 | 0.012 |
| *ROI × Context* interaction | 2.19, 50.52 | 2.70 | 0.072 | 0.105 |
| *ROI × PBO-OT* interaction | 2.45, 56.24 | 1.08 | 0.358 | 0.045 |
| *Context × PBO-OT*  interaction | 1,23 | 0.13 | 0.724 | 0.006 |
| *ROI × Context × PBO-OT* interaction | 2.26, 51.93 | 0.57 | 0.589 | 0.024 |
