# Supplementary material for: Attachment Reminders Trigger Widespread Synchrony across Multiple Brains
Source: J Neurosci. 2023 Oct 25;43(43):7213–25. doi: 10.1523/JNEUROSCI.0026-23.2023 (PMC10601370; doi:10.1523/JNEUROSCI.0026-23.2023)
Supplement: Figure 3-2 — Results of a 2 × 2 repeated-measures ANOVA (Context × PBO-OT) conducted for each of the 5 ROIs with above-threshold ISC, i.e., involved in stimulus processing. The insula and the PHG showed a significant main effect for Context. Results are Greenhouse-Geisser corrected. Abbreviations: ACC, anterior cingulate cortex; DMN, default mode network; NAcc, nucleus accumbens; OT, oxytocin; PBO, Placebo; PHG, parahippocampal gyrus;. **, p < .001. Download Figure 3-2, DOCX file. [file ns-JN-RM-0026-23-s09.docx]

**Figure 3-2.** 2×2 repeated measures ANOVA (*Context* × *PBO-OT*) for each of the preregistered ROIs above the ISC threshold.

|  | Insula | ACC | PHG | NAcc | DMN |
| --- | --- | --- | --- | --- | --- |
| *Context* main effect df(1,23) | | | | | |
| F score | 14.55 | 3.70 | 11.064 | 0.00 | 0.07 |
| *p* | 0.00** | 0.07 | 0.00** | 0.97 | 0.80 |
| Eta^2^ | 0.39 | 014 | 0.33 | 0.00 | 0.00 |
| *PBO-OT* main effect df(1,23) | | | | | |
| F score | 0.56 | 2.64 | 0.13 | 0.05 | 0.30 |
| *p* | 0.46 | 0.12 | 0.72 | 0.83 | 0.60 |
| Eta^2^ | 0.02 | 0.10 | 0.01 | 0.00 | 0.01 |
| *Context × PBO-OT* interaction df(1,23) | | | | | |
| F score | 0.10 | 0.02 | 0.46 | 0.78 | 0.03 |
| *p* | 0.75 | 0.88 | 0.50 | 0.39 | 0.87 |
| Eta^2^ | 0.00 | 0.00 | 0.00 | 0.03 | 0.00 |
