# Supplementary material for: Attachment Reminders Trigger Widespread Synchrony across Multiple Brains
Source: J Neurosci. 2023 Oct 25;43(43):7213–25. doi: 10.1523/JNEUROSCI.0026-23.2023 (PMC10601370; doi:10.1523/JNEUROSCI.0026-23.2023)
Supplement: Figure 3-3 — A 2 × 2 repeated-measures ANOVA (Context × PBO-OT) was performed to each of the 118 parcels involved in stimulus processing. The table presents 27 parcels that showed a significant main effect for Context, together with their anatomical location as defined by Neurosynth, F scores, p values, cluster sizes and cluster peak voxel. L, left; R, right. Download Figure 3-3, DOCX file. [file ns-JN-RM-0026-23-s10.docx]

**Figure 3-3.** Parcels with significant *Context* main effects results and their coordinates of activation peaks.

| Parcel | Anatomical area | F | | *p* | | Cluster size | Cluster peak voxel | | |
| --- | --- | --- | --- | --- | --- | --- | --- | --- | --- |
| number |  | (1,23) | |  |  |  | X | Y | Z |
| *Context*  main effect | | |  | |  |  |  |  |  |
| 4 | R orbitofrontal cortex | 7.75 | | 0.0065 | | 2408 | 16 | 35 | -22 |
| 27 | R premotor cortex | 38.9 | | < 0.0001 | | 4824 | 50 | -4 | 48 |
| 33 | R Motor cortex | 13.99 | | 0.00031 | | 6952 | 42 | -23 | 54 |
| 39 | Somatosensory | 11.90 | | 0.00084 | | 6704 | 21 | -33 | 70 |
| 53 | R Temporal pole | 20.40 | | < 0.0001 | | 6856 | 53 | -11 | -21 |
| 54 | R Superior temporal | 8.53 | | 0.0043 | | 2712 | 51 | -33 | 0 |
| 73 | R Visual cortex | 8.89 | | 0.0036 | | 4992 | 31 | -83 | 21 |
| 75 | R Occipital parietal cortex | 8.17 | | 0.0052 | | 6992 | 19 | -81 | 42 |
| 89 | R Midline | 10.12 | | 0.0019 | | 3360 | 9 | -22 | 45 |
| 123 | R Striatum | 7.13 | | 0.0089 | | 3624 | 13 | 21 | 0 |
| 125 | R Ventral striatum | 7.72 | | 0.0066 | | 4992 | 15 | 9 | -9 |
| 142 | L Prefrontal cortex | 7.81 | | 0.0063 | | 4088 | -29 | 55 | 3 |
| 143 | L Prefrontal cortex | 15.43 | | 0.00016 | | 4592 | -42 | 48 | -6 |
| 147 | L Dorsolateral prefrontal cortex | 15.04 | | 0.00019 | | 7040 | -46 | 29 | 27 |
| 150 | L Anterior cingulate cortex | 7.68 | | 0.0067 | | 4576 | -4 | 18 | 47 |
| 155 | L Insula | 17.17 | | < 0.0001 | | 4496 | -32 | 23 | 6 |
| 163 | L Primary somatosensory cortex | 11.58 | | 0.0009 | | 7056 | -56 | -3 | 7 |
| 165 | L Premotor cortex | 6.91 | | 0.010 | | 5448 | -45 | 0 | 50 |
| 171 | L Motor cortex | 8.24 | | 0.0050 | | 6728 | -50 | -23 | 42 |
| 181 | L Somatosensory cortex | 13.19 | | 0.00046 | | 6840 | -59 | -25 | 22 |
| 184 | L Inferior [parietal](https://neurosynth.org/analyses/terms/parietal) cortex | 28.20 | | < 0.0001 | | 10072 | -53 | -43 | 39 |
| 188 | L Temporal pole | 9.92 | | 0.0022 | | 5992 | -49 | 7 | -15 |
| 191 | L Auditory cortex | 7.60 | | 0.007 | | 6872 | -58 | -29 | 4 |
| 192 | L Superior temporal | 12.93 | | 0.00052 | | 7544 | -57 | -47 | 6 |
| 217 | L [Paralimbic](https://neurosynth.org/analyses/terms/paralimbic) | 17.48 | | < 0.0001 | | 2368 | -23 | -41 | 20 |
| 230 | L Parahippocampal gyrus | 13.98 | | 0.00031 | | 2512 | -32 | -40 | -3 |
| 246 | L Cerebellum | 9.75 | | 0.0023 | | 6752 | -42 | -63 | -46 |
