# Supplementary material for: Attachment Reminders Trigger Widespread Synchrony across Multiple Brains
Source: J Neurosci. 2023 Oct 25;43(43):7213–25. doi: 10.1523/JNEUROSCI.0026-23.2023 (PMC10601370; doi:10.1523/JNEUROSCI.0026-23.2023)
Supplement: Figure 4-1 — Non-significant brain–behavior Pearson's correlation results in ROIs. NAcc, Nucleus accumbens; PHG, parahippocampal gyrus; DMN, default mode network. Download Figure 4-1, DOCX file. [file ns-JN-RM-0026-23-s13.docx]

**Figure 4-1.** Non-significant brain-behavior correlation results in ROIs.

|  | ***r*** | ***p*** |
| --- | --- | --- |
| Insula | 0.32 | 0.054 |
| NAcc | 0.07 | 0.662 |
| PHG | 0.08 | 0.631 |
| DMN | 0.24 | 0.152 |
